# Nuclear translocation of MTL5 from cytoplasm requires its direct interaction with LIN9 and is essential for male meiosis and fertility

**DOI:** 10.1371/journal.pgen.1009753

**Published:** 2021-08-13

**Authors:** Xingxia Zhang, Ming Li, Xiaohua Jiang, Hui Ma, Suixing Fan, Yang Li, Changping Yu, Jianze Xu, Ranjha Khan, Hanwei Jiang, Qinghua Shi

**Affiliations:** First Affiliated Hospital of USTC, Hefei National Laboratory for Physical Sciences at Microscale, School of Basic Medical Sciences, Division of Life Sciences and Medicine, CAS Center for Excellence in Molecular Cell Science, University of Science and Technology of China, Hefei, China; University of Nevada School of Medicine, UNITED STATES

## Abstract

Meiosis is essential for the generation of gametes and sexual reproduction, yet the factors and underlying mechanisms regulating meiotic progression remain largely unknown. Here, we showed that MTL5 translocates into nuclei of spermatocytes during zygotene-pachytene transition and ensures meiosis advances beyond pachytene stage. MTL5 shows strong interactions with MuvB core complex components, a well-known transcriptional complex regulating mitotic progression, and the zygotene-pachytene transition of MTL5 is mediated by its direct interaction with the component LIN9, through MTL5 C-terminal 443–475 residues. Male *Mtl5*^*c-mu/c-mu*^ mice expressing the truncated MTL5 (p.Ser445Arg fs*3) that lacks the interaction with LIN9 and is detained in cytoplasm showed male infertility and spermatogenic arrest at pachytene stage, same as that of *Mtl5* knockout mice, indicating that the interaction with LIN9 is essential for the nuclear translocation and function of MTL5 during meiosis. Our data demonstrated MTL5 translocates into nuclei during the zygotene-pachytene transition to initiate its function along with the MuvB core complex in pachytene spermatocytes, highlighting a new mechanism regulating the progression of male meiosis.

## Author summary

Meiosis is essential for spermatogenesis and male fertility. However, the factors regulating the progression of meiosis remain largely unknown. We reported the testis specific protein MTL5 translocated into the nuclei of spermatocytes at the zygotene-pachytene transition by direct interaction with LIN9, which is an essential component of MuvB core complex, to promote meiotic progression beyond the pachytene stage. We also showed that MTL5 pulls down MYBL1 and all of the MuvB core complex (except LIN54) in spermatocytes. Given the known role of the MuvB core complex as a cell cycle regulator in mitotic cells, we suggested that MTL5 promotes meiotic progression along with the MuvB core complex to ensure male fertility. Our results indicated a novel function of the MuvB complex in male meiosis and also shed light on the master regulator proteins that control meiotic progression at the pachytene stage. MTL5 is a novel and germ-cell specific regulator of cell cycle progression to function at a specific stage by nuclear translocation in meiosis.

## Introduction

Infertility, which affects roughly 15% of reproductive-aged couples worldwide, is emerging as both a serious medical issue as well as a major social problem [1–4]. Although artificial assisted reproductive technology can enable an increasing number of infertile couples to give birth to their own children, men with infertility caused by meiotic dysfunction do not benefit from these techniques due to the lack of gametes [5,6]. Thus, studies regarding genes that are essential for meiotic progression help increase our fundamental understanding of the mechanisms controlling meiosis [7,8].

Meiotic prophase I, which contains a series of meiosis-specific events, including meiotic recombination and synapsis, is equivalent to the G2 phase of the cell cycle for somatic cells [9–14]. It takes longer (lasting around 13 days in mice) than mitotic cells to guarantee the orderly progression of these meiotic events. The meiotic G2 phase (meiotic prophase I) is divided into a series of sub-stages, including leptotene, zygotene, pachytene, diplotene, and diakinesis, according to the chromosome configurations and structure [15]. Its progression is strictly regulated by different checkpoints that monitor the proper occurrence of meiotic events, which lead to meiotic arrest (usually at pachytene stage) when meiotic errors take place. This suggests the existence of a cell cycle regulator that promotes or blocks meiotic progression under the regulation of meiotic checkpoints.

The MuvB core complex, which consists of LIN9, LIN54, LIN37, LIN52, and RBBP4, is a well-known cell cycle regulator that serves essential roles during various phases of mitosis cell cycle by binding and directing different key transcription factors to the promoters of cell cycle genes [16,17]. During the G0 and early G1 phase, the MuvB core interacts with E2F4/5, DP1/2, and p130/p107 to form the DREAM complex (dimerization partner (DP), RB-like, E2F and MuvB) that represses the expression of genes. These co-factors are then released from the MuvB core complex to allow the recruitment of B-MYB to MuvB core complex (MMB complex) in the S and early G2 phase, promoting G2/M transition. This complex further recruits FOXM1 in the late G2 phase to activate G2/M genes [16,17]. However, it is unknown whether and how this complex functions during meiotic prophase I.

MTL5 (Metallothionein-like 5, also known as *Tesmin*) is a testis-specific metallothionein-like protein which contains tandem cysteine-rich regions (CRC domain, the DNA-binding domain), and it shares sequence similarity with LIN54 [18]. Mouse *Mtl5* gene locates on chromosome 19 and it has two transcript isoforms, with the longer and more common transcript, encoding a 475 amino acids (aa) protein, specifically expressed in mouse testes [19]. As a testis-specific metallothionein, *Mtl5* mRNA expression is first detected at 8 days post-partum (dpp), which is consistent with the initiation of meiosis [20]. Despite of the early expression of mRNA, homozygous *Mtl5* deletion in mice does not cause any obvious defects in the leptotene and zygotene stages, but results in male infertility due to spermatogenic arrest at the pachytene/diplotene stage [21].

Intriguingly, immunocytochemical analysis of MTL5 localization in mouse testes by Matsuura et al. showed that it shuttles between the cytoplasm and nuclei of spermatocytes during spermatogenesis [22]. Sutou et al. found that MTL5 mainly locates in the cytoplasm of spermatocytes in stages I-IX tubules, which corresponds to early and mid-pachytene spermatocytes, but then translocates into the nuclei of spermatocytes at the late pachytene or diplotene stages, which are present in stage X-XII tubules [19]. Taken together, these reports suggested a possible relationship between the nuclear translocation of MTL5 and its function.

To uncover the mechanism and biological significance of the translocation of MTL5 from the cytoplasm to nucleus, we first studied its localization in mouse testes and found that MTL5 translocates to the nucleus around zygotene-pachytene transition. We then showed that MTL5 interacts with all the MuvB core complex components except LIN54 in spermatocytes and transportes into the nuclei directly by LIN9 in cultured cells. We also showed that the nuclear translocation of MTL5 is carried out by LIN9, the subunit of the MuvB core complex which is well-known in the DREAM complex [23,24], through its direct interaction with the C-terminal 443–475 aa region of MTL5. A truncation mutation of MTL5 (p.Ser445Arg fs*3), which generated the *Mtl5*^*c-mu/c-mu*^ mouse line, eliminated its interaction with LIN9, and consequently led to cytoplasmic retention of MTL5 in pachytene spermatocytes. Male mice homozygous for the mutation showed spermatogenesis arrest at pachytene and azoospermia, exactly the same phenotype as in *Mtl5* knockout (*Mtl5*^*-/-*^) mice, indicating that MTL5 is transported into the nuclei by LIN9 during zygotene-pachytene transition to promote meiotic progression beyond pachytene stage.

## Results

### MTL5 shifts from the cytoplasm to nuclei of spermatocytes during zygotene-pachytene transition

Previous studies in mice reported that MTL5 localized to the cytoplasm or nucleus in a spermatogenic cell type-specific manner [19], but the stage at which MTL5 translocates from cytoplasm into nuclei of spermatocytes during spermatogenesis was not fully resolved. To determine the exact stage in which MTL5 translocates from cytoplasm to nucleus, we analyzed the expression and localization patterns of MTL5 during spermatogenesis of mice in detail. MTL5 protein is expressed specifically in mouse testes (S1A Fig) and was first detected at 10 dpp in mouse testes (S1B Fig), which corresponds to the leptotene and zygotene stages of the first wave of spermatogenesis. Consistent with this finding, RT-PCR on isolated spermatogenic cells showed that *Mtl5* expression was initially detected in leptotene and zygotene spermatocytes, then increased sharply in pachytene and diplotene spermatocytes, and finally disappeared in spermatids (S1C Fig).

**Fig 1:**
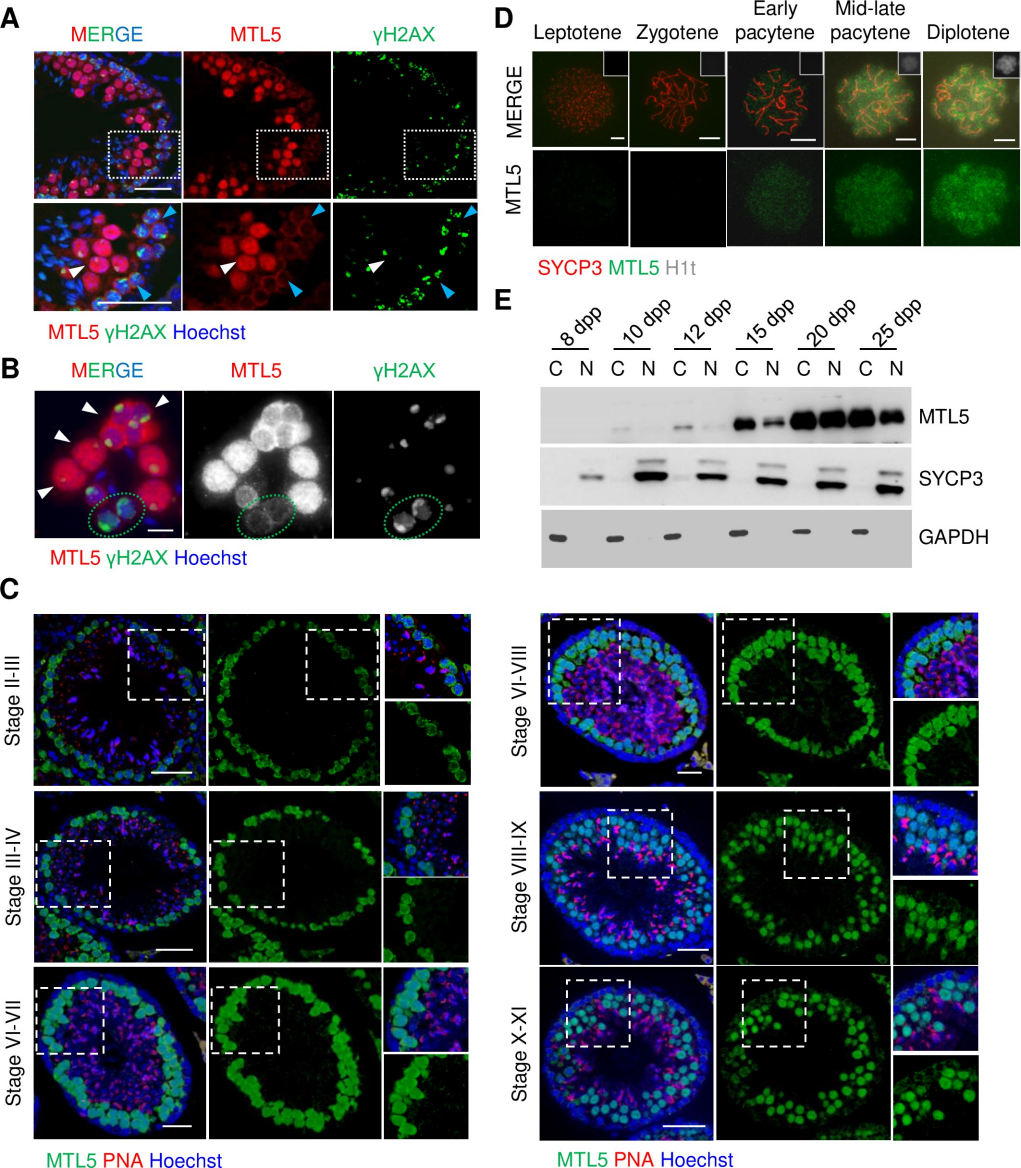
The dynamic localization of MTL5 in spermatocytes of meiotic prophase I. (A) Immunostaining of MTL5 (red) and γH2AX (green) in testicular sections of 10-week-old wild-type mice. Nuclei were counterstained with Hoechst 33342 (blue). Enlarged views (bottom) are indicated by white boxes (top). White arrowheads indicate pachytene or diplotene spermatocytes. Blue arrowheads indicate leptotene or zygotene spermatocytes. Scale bars, 50 μm. (B) Immunostaining for MTL5 (red) and γH2AX (green) in smeared testicular cells of 10-week-old wild-type mice for MTL5 (red) and γH2AX (green). Nuclei were counterstained with Hoechst 33342 (blue). White arrowheads indicate pachytene or diplotene spermatocytes. Green dotted ovals indicate zygotene spermatocytes. Scale bars, 10 μm. (C) Immunostaining of MTL5 (green) and PNA (red) in testicular sections of 10-week-old wild-type mice. Nuclei were counterstained with Hoechst 33342 (blue). Enlarged views are indicated by white boxes. Scale bars, 50 μm. (D) Immunostaining for SYCP3 (red), H1t (gray), and MTL5 (green) in spread spermatocytes of 10-week-old wild-type mice. Scale bars, 10 μm. (E) Western blot analyses were performed using cytoplasmic and nuclear protein extracts from 8, 10, 12, 15, 20, and 25 days post-partum (dpp) mouse testes. GAPDH and SYCP3 served as cytoplasmic and nuclear controls, respectively.

The localization of MTL5 was determined by the co-staining of MTL5 and γH2AX on either testicular sections or smeared testicular cells (Fig 1A and 1B). The MTL5 signal was first observed in cytoplasm of leptotene and zygotene spermatocytes, which showed dispersed γH2AX signals, and became stronger in the nuclei of pachytene and diplotene spermatocytes, while no MTL5 signals were detected in pre-meiotic and Sertoli cells (Fig 1A and 1B). To detect the dynamic nuclear localization of MTL5 in pachytene/diplotene spermatocytes, immunostaining of MTL5 and PNA were performed on testicular sections from 10-week-old wild-type mouse and seminiferous tubule stages were distinguished according to the morphology of PNA staining (Fig 1C). The localization of MTL5 was observed in both cytoplasm and nuclei of the early pachytene spermatocytes in stage II, and then gradually accumulated in the nuclei of pachytene/diplotene spermatocytes from stage III to VIII (Fig 1C). Almost no cytoplasm localization of MTL5 was observed in pachytene/diplotene spermatocytes from tubules after stage VIII (Fig 1C). Then, we stained H1t, which is expressed in germ cells after the mid-pachytene stage, to further detect the dynamics of MTL5 nuclear localization on spermatocyte spreads (Fig 1D). MTL5 signals were first observed in the nuclei from early-pachytene spermatocytes, and became stronger in the nuclei of the mid-late pachytene and diplotene spermatocytes (Fig 1D).

To further confirm the translocation of MTL5, we separated the cytoplasm and nuclei of spermatocytes from 8 dpp to 25 dpp mice and determined the level of MTL5 by Western blot (Fig 1E). Consistent with the immunofluorescence localization, no MTL5 was detected in either cytoplasmic or nuclear lysates of 8 dpp testes, but was subsequently observed in cytoplasm lysate of 10 dpp testes and then for the first time in nuclei of spermatocytes in 12 dpp testes (Fig 1E). With the progression of the spermatogenic wave, MTL5 protein levels gradually increased in both cytoplasmic and nuclear lysates of spermatocytes from 15 dpp onward (Fig 1E).

These results indicate that MTL5 expression is restricted to the cytoplasm of spermatocytes during the leptotene and zygotene stages, and then starts to translocate into the nuclei of germ cells after the zygotene-pachytene transition.

### MTL5 is transported into the nuclei by the MuvB core complex component LIN9

**Fig 2:**
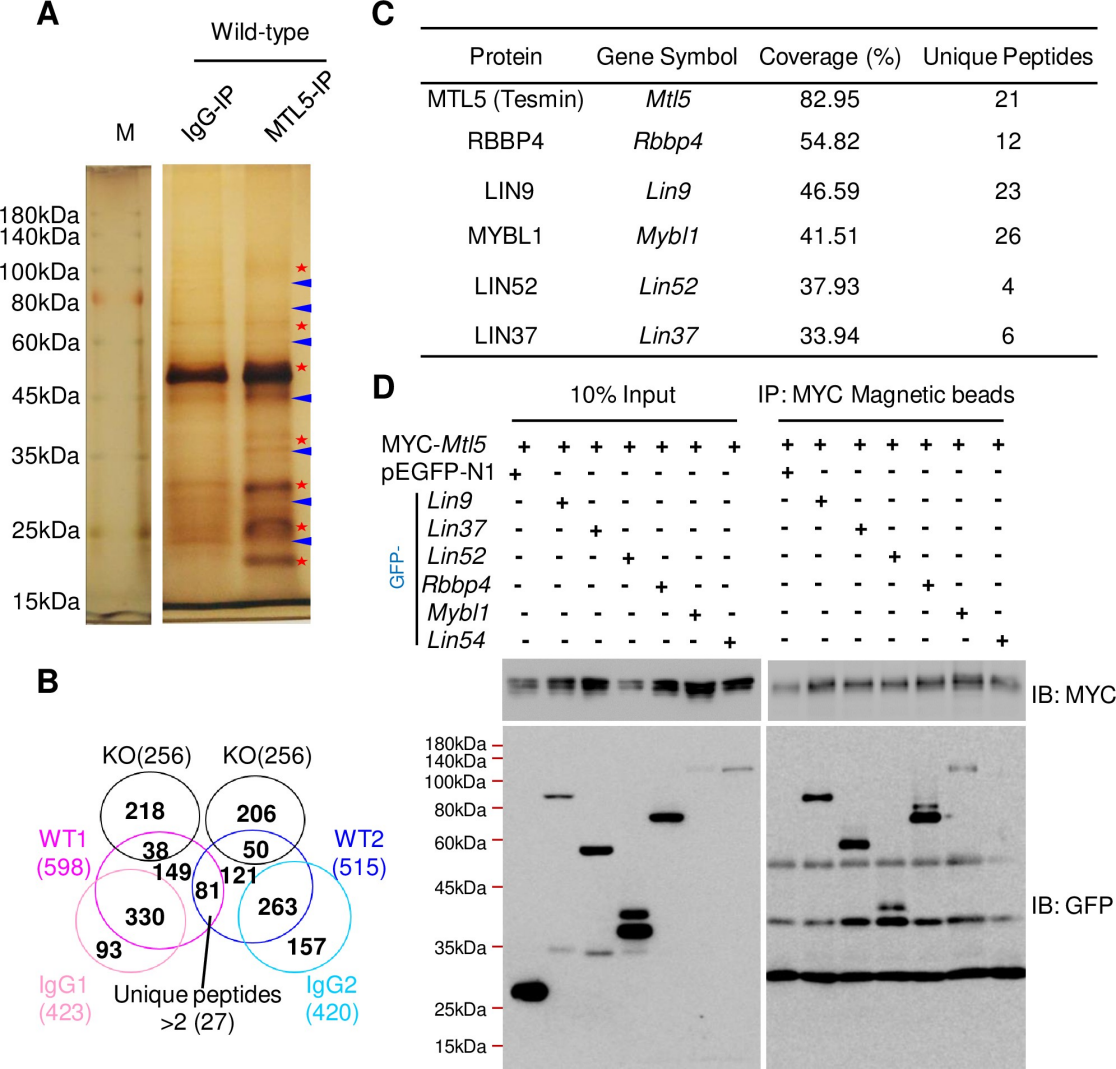
MTL5 interacts with most components of the MuvB core complex. (A) Silver staining of the samples immunoprecipitated with MTL5 antibody (epitope from 221 aa to 475 aa) from pachytene and diplotene spermatocytes of 10-week-old wild-type mouse after SDS-PAGE. Rabbit IgG served as a negative control. Red stars mark the different bands between IgG and MTL5 antibody group. Blue arrowheads indicate the unspecific bands. M indicates protein marker. (B) Identification of proteins interacting with MTL5 by IP-MS followed by subtractive analysis of three proteomic data sets with two biological repetitions. Proteins with at least three unique peptides in wild-type groups, but absent in both IgG and knockout (KO) groups, were considered as candidate MTL5-interactive proteins. Numbers in the brackets indicate the number of proteins. (C) Rank of the top candidates, from high to low, sorted by the coverage (%). The top 6 candidates are shown. (D) Western blot detection of the interaction between MTL5 (fused with a MYC tag) and each of MuvB core complex components or MYBL1 (fused with a GFP tag) in HEK293T cells after co-immunoprecipitation with MYC-tag magnetic beads. pEGFP-N1 was served as the negative control. IP, immunoprecipitation. IB, immunoblotting.

Based on protein structure analysis, MTL5 does not have a clear nuclear localization sequence (NLS), strongly suggesting that other proteins mediate or contribute to its transport into nuclei. To identify the proteins that help the nuclear translocation of MTL5, we thus performed immunoprecipitation with an antibody against MTL5 in the pachytene and diplotene spermatocytes enriched from 10-week-old wild-type mice testes using the STA-PUT method [25], followed by mass spectrometry analysis (IP-MS) (Fig 2A and 2B). In total, 27 putative candidate MTL5 interacting proteins which contain at least 3 unique peptides were identified (S1 Table). Interestingly, besides MTL5 and MYBL1, 4 out of 5 components of MuvB core complex, including RBBP4, LIN9, LIN52, and LIN37, appeared at the top of candidate list ranked by coverage (%) (Fig 2C). Notably, MYBL1 is the homologue of MYBL2, which has been experimentally proven to interact with MuvB core complex in somatic cells [26–28,16]. To confirm the interaction of MTL5 with the MuvB core complex, we performed co-immunoprecipitation (co-IP) in lysates of HEK293T cells after co-expressing MTL5 with all the components of MuvB core complex (including LIN9, LIN37, LIN52, RBBP4, and LIN54) as well as MYBL1. As expected, the co-IP results showed that MTL5 interacted with MYBL1 and all MuvB core complex components (Fig 2D) except LIN54 which is particular lowly expressed in testis (S2 Fig).

To determine which component(s) of MuvB core complex interact(s) directly with MTL5, we examined the interactions between MTL5 (prey, transformed into the Y187 yeast haploid strain) and LIN9, LIN37, LIN52, RBBP4, or MYBL1 (baits, transformed into the AH109 yeast haploid strain) by using yeast two-hybrid (Y2H) screening. Only the transformed AH109 and Y187 yeast haploid cells with MTL5 and LIN9 or MTL5 and RBBP4 could grow on all selective medium plates (S3 Fig), indicating that MTL5 interacts with LIN9 and RBBP4 directly.

**Fig 3:**
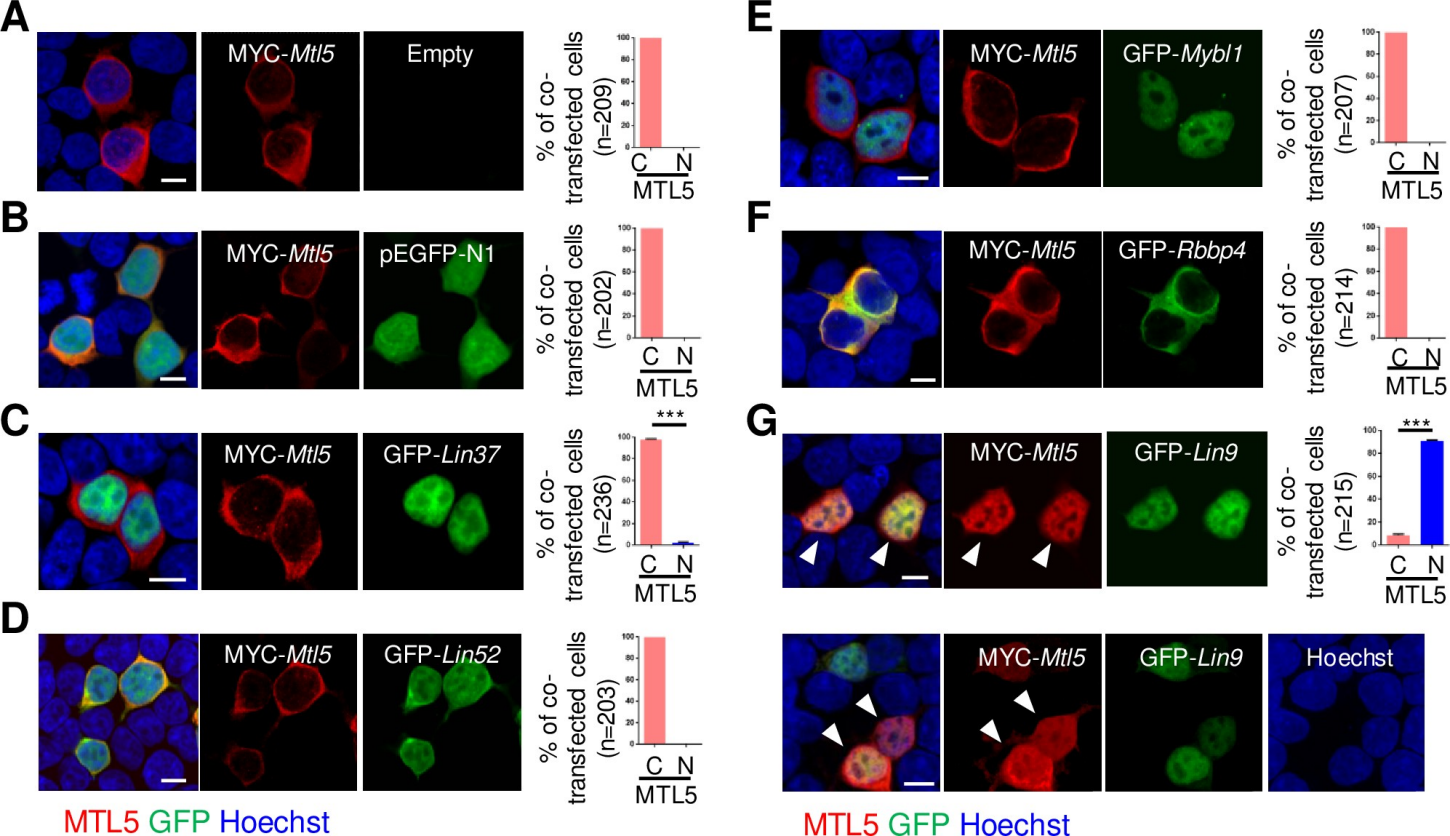
MTL5 is transported into the nuclei of cultured HEK293T cells by LIN9. HEK293T cells were transiently transfected with plasmids encoding (A) MYC-*Mtl5*, (B) MYC-*Mtl5* and pEGFP-N1, (C) MYC-*Mtl5* and GFP-*Lin37*, (D) MYC-*Mtl5* and GFP-*Lin52*, (E) MYC-*Mtl5* and GFP-*Mybl1*, (F) MYC-*Mtl5* and GFP-*Rbbp4*, or (G) MYC-*Mtl5* and GFP-*Lin9*. The localization of proteins are observed by immunostaining with antibodies against MTL5 (red) and GFP (green) antibodies, and imaged by a Nikon confocal laser scanning microscope system. Whit arrowheads indicate the co-localization of MTL5 and LIN9. The cells with nuclear (N) or cytoplasmic (C) localization of MTL5 were counted and “n” in the brackets represents the number of co-transfected cells that were analyzed. Data are presented as mean ± SEM from three independent experiments. P values were analyzed by *t-*test. ***p<0.001. Scale bars, 10 μm.

Based on the above findings, we propose that the nuclear translocation of MTL5 from cytoplasm is mediated by binding to LIN9 or/and RBBP4. We thus examined the localization of MTL5 in HEK293T cells co-transfected with plasmids encoding MYC-*Mtl5* and plasmids encoding GFP-*Lin9* or GFP-*Rbbp4*, respectively (for the controls, GFP-*Lin37*, GFP-*Lin52*, or GFP-*Mybl1* encoded plasmids were also co-transfected with MYC-*Mtl5* constructs respectively). MTL5 was diffusely localized in the cytoplasm of HEK293T cells when transfected with MYC-*Mtl5* alone or with the pEGFP-N1 vector (Fig 3A and 3B). As expected, the cytoplasmic localization of MTL5 was also observed in cells with MTL5 co-expressed with LIN37, LIN52, or MYBL1, because no direct interaction of MTL5 with them was detected in Y2H screening (Fig 3C–3E). What is more, RBBP4, a previously identified direct interacting partner of MTL5, only showed a cytoplasmic localization and failed to transport MTL5 into the nucleus (Fig 3F). Instead, co-expression with LIN9, another MTL5-interacting protein, caused a dramatic redistribution of MTL5 from cytoplasm to nuclei in HEK293T cells (Fig 3G), indicating that LIN9 may serve as the key factor for MTL5 nuclear translocation.

### LIN9 transports MTL5 into nuclei by the direct interaction with the C-terminal 443–475 amino acids of MTL5 in cultured somatic cells

**Fig 4:**
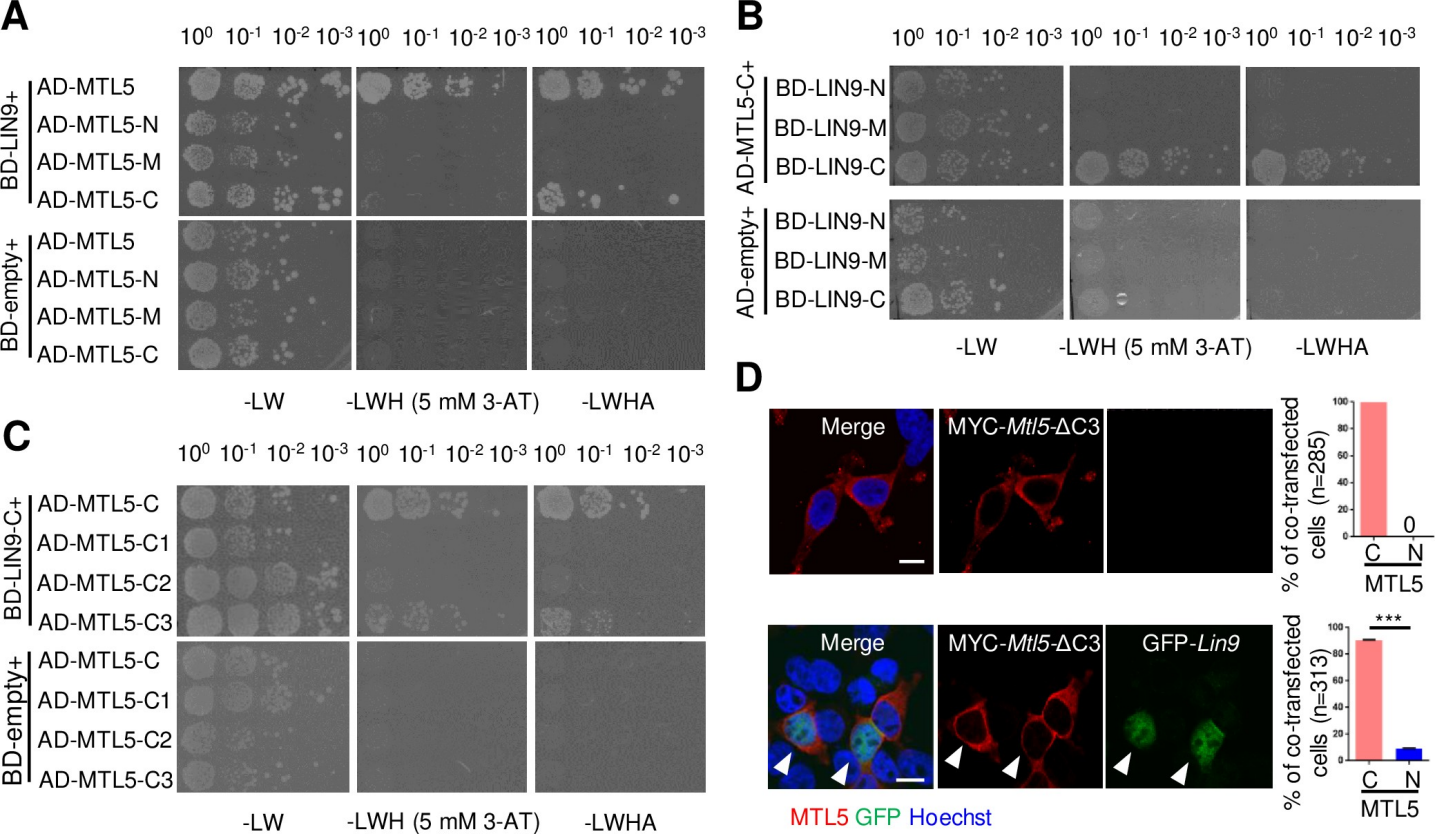
LIN9 directly interacts with MTL5 C-terminal residues 443–475 for transport of MTL5 into the nucleus. (A) The Y2H was used to assess the interaction of truncated MTL5 (MTL5-N, MTL5-M, and MTL5-C) with full length LIN9, respectively. MTL5-N contains residues 1–250 aa;MTL5-M contains residues 251–370 aa;MTL5-C indicates contains 371–475 aa. (B) Y2H assessment of interactions between of truncated LIN9 (LIN9-N, LIN9-M, and LIN9-C) with AD-MTL5-C (371–475 aa) respectively. LIN9-N contains residues 1–150 aa;LIN9-M contains residues 151–340 aa;LIN9-C contains residues 341–559 aa. (C) The interactions of AD-MTL5-C1 (371–418 aa), AD-MTL5-C2 (419–442 aa), and AD-MTL5-C3 (443–475 aa) with BD-LIN9-C (341–559 aa) or BD-empty (control) was assessed by Y2H, respectively. The interaction in (A-C) was tested by growth of the yeast cells on double (SD-Leu/Trp, -LW), triple (SD-His/Leu/Trp with 5 mM 3-AT, -LWH) or quadruple dropout medium plates (SD-Ade/His/Leu/Trp, -LWHA) at different dilutions. (D) HEK293T cells were transiently co-transfected with plasmids encoding MYC-*Mtl5*-ΔC3 (C-terminal 443–475 aa residues were deleted) and GFP-LIN9. The localization of LIN9 and mutant MTL5 was detected by immunostaining with antibodies against GFP (green) and MTL5 (red), respectively. The nuclei (blue) of HEK293T cells were counterstained with Hoechst 33342. The cells with nuclear (N) or cytoplasmic (C) localization of MTL5 were counted and “n” in the brackets represent the number of (co-)transfected cells analyzed. White arrowheads indicates the localization of truncated MTL5 and LIN9 in HEK293T cells. Data are presented as mean ± SEM of three independent replicates. P values were detected by Student’s *t*-test. ***p<0.001. Scale bar, 10 μm.

To determine which regions of MTL5 are responsible for direct interaction with LIN9, and subsequent importation into nuclei, we performed Y2H screening assays using truncated variants of MTL5 to identify which exhibited direct interactions with LIN9. MTL5 consists of 475 aa and contains a CRC domain in the region from 263 aa to 370 aa, as well as two predicted helix domains (from 420 aa to 439 aa and from 444 aa to 468 aa) in the C terminus. Thus, we cloned the fragments of MTL5-N (1–250 aa), MTL5-M (251–370 aa) that contains the CRC domain, and MTL5-C (371–475 aa) containing the predicted helix domains, into pGADT7 expression vectors to produce AD-MTL5-N (1–250 aa), AD-MTL5-M (251–370 aa), and AD-MTL5-C (371–475 aa), respectively (S4A Fig). These vectors were transformed into the Y187 yeast haploid strain and then hybridized with BD-LIN9, which was transformed into the AH109 yeast haploid strain. Only the transformed Y187 and AH109 yeast haploid cells transformed with AD-MTL5-C and BD-LIN9 could grow on both double and quadruple dropout medium plates (Fig 4A), suggesting that the region mediating direct interaction between MTL5 and LIN9 is located in the MTL5 C-terminal 371–475 aa. We also cloned the fragments of LIN9 (S4B Fig) into the pGBKT7 expression vector, performed Y2H screening assays with AD-MTL5-C, and found that the C-terminus (341–559 aa) region of LIN9 exhibited direct interaction with the C terminus of MTL5-C (371–475 aa) (Fig 4B).

To map more precisely the region(s) of MTL5 that interact with LIN9, we further cloned three fragments of MTL5 C-terminal 371–475 aa into pGADT7 expression vectors to generate AD-MTL5-C1 (371–418 aa), AD-MTL5-C2 (419–442 aa), and MTL5-C3 (443–475 aa) (S4A Fig). Only the transformed Y187 and AH109 yeast haploid cells with AD-MTL5-C3 and BD-LIN9-C grew on all selective medium plates (Fig 4C), suggesting that the C-terminal 443–475 aa of MTL5, which contains the second predicted helix domain from 444 aa to 468 aa, is essential for its direct interaction with LIN9.

Given that the C-terminal 443–475 aa of MTL5 is necessary for its direct interaction with LIN9, we hypothesized that this direct interaction might be essential for the nuclear translocation of MTL5 from the cytoplasm. To test this, we generated a MYC-*Mtl5*-ΔC3 vector that encodes a truncated MTL5 lacking the C-terminal 443–475 aa (S4C Fig), and then transfected it into HEK293T cells with the GFP-tagged mouse LIN9 (GFP-*Lin9*) vectors. It is shown that LIN9 failed to transport MTL5-ΔC3 into the nuclei of transfected cells (Fig 4D), but was able to transport the truncated MTL5 with deletion in other regions into nuclei (S5 Fig). These results further indicate that the C-terminal 443–475 aa of MTL5 is essential for its translocation from the cytoplasm to the nucleus, mediated by the direct interaction with LIN9.

### Translocation of MTL5 into the nuclei of spermatocytes requires the MTL5 C-terminus and is essential for meiotic progression and sperm production

Given the function of LIN9 to translocate MTL5 into the nuclei of somatic cells, we hypothesized that LIN9 could be essential for the nuclear translocation of MTL5 in spermatocytes. To test this, we first examined whether LIN9 showed nuclear localization in pachytene spermatocytes. The localization of LIN9 was detected in 10-week-old mouse testes by immunofluorescence staining, and we found that LIN9 signals were observed in the nuclei of Sertoli cell, spermatogonia and pachytene/diplotene spermatocytes, while no obvious LIN9 signals were observed in leptotene/zygotene spermatocytes (S6A Fig). Furthermore, LIN9 protein was first detected in spermatocytes around 12 dpp, which is consistent with its appearance in pachytene spermatocytes when nuclear translocation of MTL5 occurs (S6B Fig).

**Fig 5:**
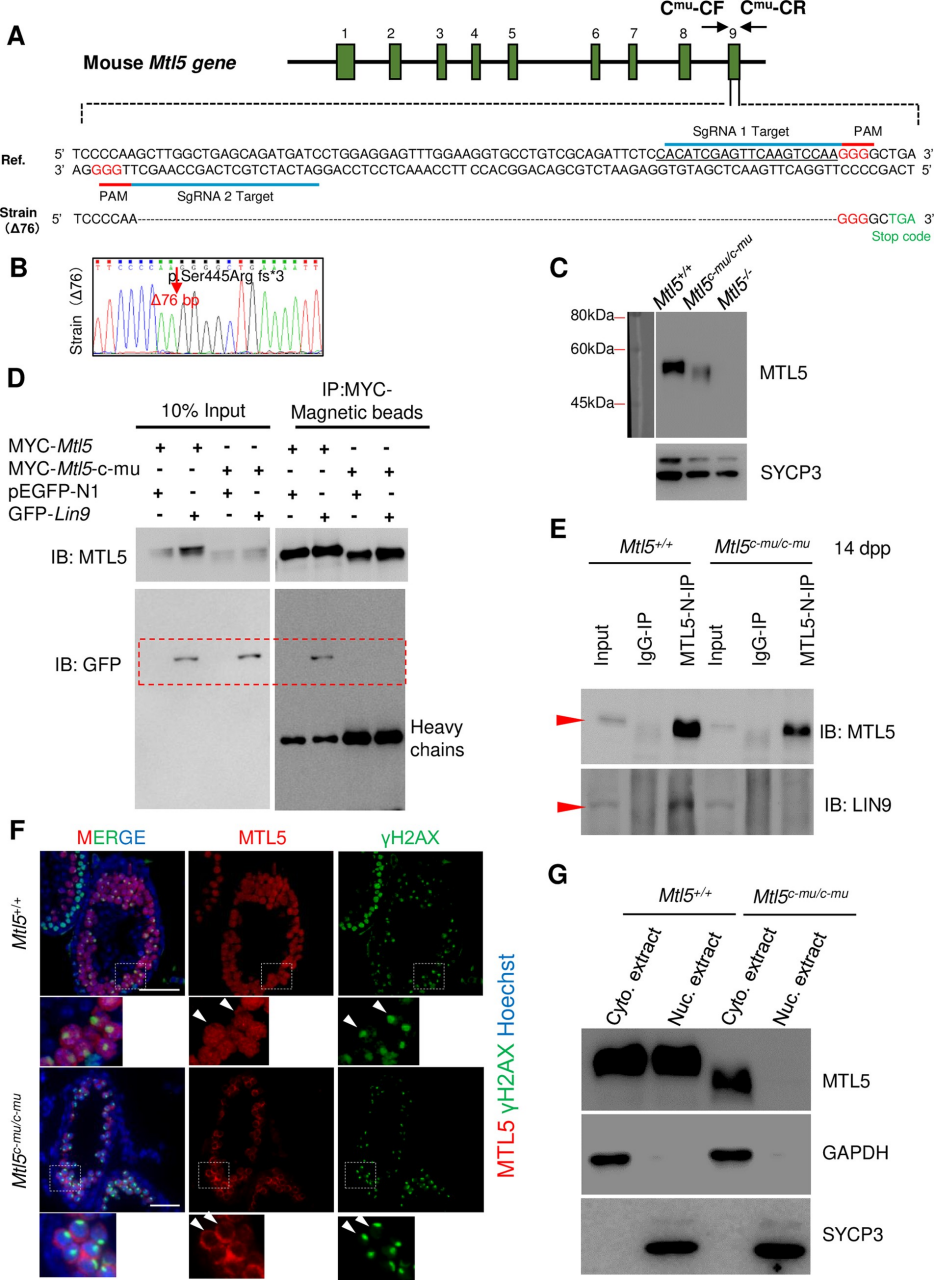
The C-terminus deleted MTL5 (Ser445Arg fs*3) exhibits abolished interaction with LIN9 and fails to be translocated into nuclei of spermatocytes in *Mtl5*^*c-mu/c-mu*^ mice. (A) Diagram (top) and nucleotide sequence (bottom) of the mouse *Mtl5* locus from the Ensemble database (ENSMUST00000025840.15) with highlighted coding exons (green) and the TAG (light green) premature stop codon of the modified transcript (bottom). PAM, protospacer adjacent motif. sgRNA, single-guide RNA. C^mu^-CF and C^mu^-CR represent the genotyping primers of *Mtl5* mutant mice. (B) The mutant mouse strain with a 76 bp deletion from position 1335 to 1410 in exon 9 (*Mtl5*^*c-mu/c-mu*^) was confirmed by Sanger sequencing. The position of the deletion site in the truncated MTL5 (p.Ser445Arg fs*3) produced by *Mtl5*^*c-mu/c-mu*^ is indicated by the arrow. (C) Western blot analysis of MTL5 levels in testes from *Mtl5*^*+/+*^, *Mtl5*^*c-mu/c-mu*^, and *Mtl5*^*-/-*^ mice. SYCP3 served as loading control to indicate the comparable composition of cell populations. (D) Western blot analysis of the interaction between truncated MTL5 (p.Ser445Arg fs*3) and LIN9 in HEK293T cells after co-transfection followed by co-immunoprecipitation with MYC-Magnetic beads. MYC-*Mtl5*-c-mu encoding the truncated MTL5 (p.Ser445Arg fs*3). pEGFP-N1 served as the negative control. IP, immunoprecipitation. IB, immunoblotting. (E) Western blot analysis of the interaction between truncated MTL5 (p.Ser445Arg fs*3) and LIN9 in 14 dpp *Mtl5*^*+/+*^ and *Mtl5*^*c-mu/c-mu*^ mouse testes after co-immunoprecipitation with MTL5-N antibody. IP, immunoprecipitation. IB, immunoblotting. Red arrowheads indicate the expected bands. (F) Immunostaining of MTL5 (red) and γH2AX (green) on testicular sections from 4-week-old *Mtl5*^*+/+*^ and *Mtl5*^*c-mu/c-mu*^ mice. Nuclei were counterstained with Hoechst 33342 (blue). White arrowheads indicate the pachytene spermatocytes with intact sex-body. Scale bars, 50 μm. (G) Western blot analyses of MTL5 and truncated MTL5 (p.Ser445Arg fs*3) using cytoplasmic and nuclear protein extracts from 4-week-old *Mtl5*^*+/+*^ and *Mtl5*^*c-mu/c-mu*^ mouse testes. GAPDH and SYCP3 served as cytoplasmic and nuclear controls, respectively.

To further test whether the interaction between MTL5 and LIN9 is also essential for the nuclear translocation of MTL5 in spermatocytes, we generated MTL5 C-terminal truncated mice (*Mtl5*^*c-mu/c-mu*^), which produced a truncated protein p.Ser445Arg fs*3. This mutation is predicted to ablate the second potential helix domain within C-terminal 443–475 aa of MTL5, which abolished LIN9 interaction (Fig 5A and 5B). At the same time, we obtained the MTL5 knockout mice (*Mtl5*^*-/-*^) (S7 Fig). Indeed, we detected the presence of truncated MTL5 at the expected size in *Mtl5*^*c-mu/c-mu*^ testes by Western blot using the anti-MTL5 N-terminal antibody (epitope 1–220 aa) (Fig 5C). The truncated MTL5 (p.Ser445Arg fs*3) abolished its interaction with LIN9 in HEK293T cells (Fig 5D). The disturbed interaction between MTL5 and LIN9 was also confirmed in 14 dpp *Mtl5*^*c-mu/c-mu*^ mice testes (Fig 5E). As expected, we did not see any MTL5 signals present in the nuclei of pachytene spermatocytes in *Mtl5*^*c-mu/c-mu*^ testes examined using immunofluorescence staining (Fig 5F). Consistent with the immunofluorescence results, no MTL5 protein was detected in the nuclear extract of *Mtl5*^*c-mu/c-mu*^ mice (Fig 5G). Taken together, these results further indicate that the C-terminal 445–475 aa of MTL5 is responsible for MTL5 nuclear translocation in spermatocytes and highlights an essential role of the LIN9-MTL5 interaction in MTL5 nuclear transportation in spermatocytes during spermatogenesis.

**Fig 6:**
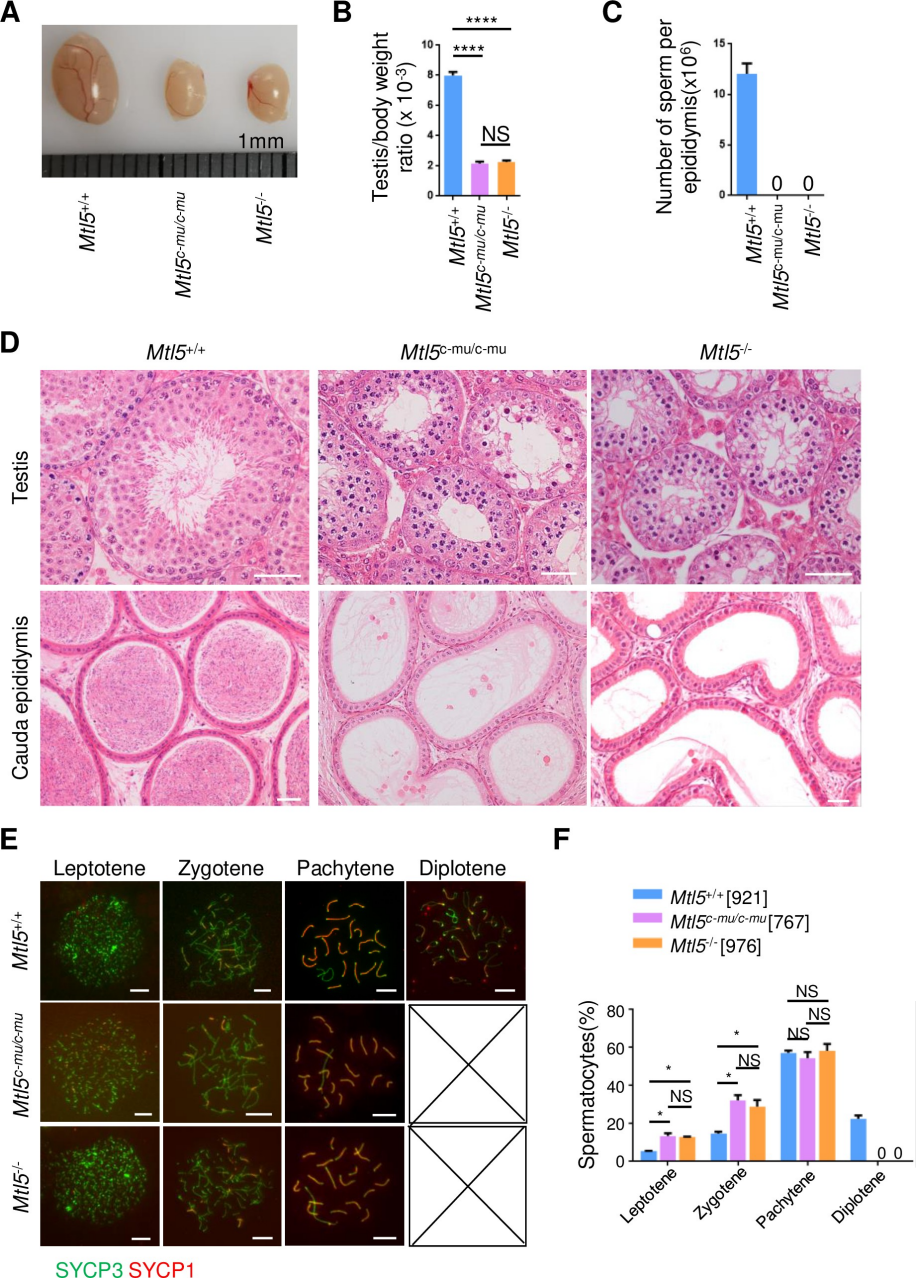
MTL5 C-terminal deletion leads to spermatogenesis arrest at pachytene stage in *Mtl5*^*c-mu/c-mu*^ mice. (A) Testis morphology, (B) Ratios of testis/body weight and (C) Epididymal sperm counts of 10-week-old *Mtl5*^*+/+*^, *Mtl5*^*-/-*^, and *Mtl5*^*c-mu/c-mu*^ mice. (D) H&E staining of testicular and epididymal sections from 10-week-old mice. Scale bars, 50 μm. (E) Immunostaining for SYCP3 (green) and SYCP1 (red) in surface-spread spermatocytes from 10-week-old *Mtl5*^*+/+*^, *Mtl5*^*-/-*^, and *Mtl5*^*c-mu/c-mu*^ mice. Scale bars, 10 μm. (F) Percentages of spermatocytes at sub-stages of meiotic prophase I. The numbers in the brackets indicate the number of spermatocytes counted. Data are presented as mean ± SEM from at least four mice. P values were analyzed by One-way ANOVA. *p<0.05;****p<0.0001;NS, p>0.05.

To determine whether the nuclear translocation of MTL5 is required for its function *in vivo*, we examined the spermatogenesis and meiosis in *Mtl5*^*c-mu/c-mu*^ mice, and compared with those in *Mtl5*^*+/+*^ and *Mtl5*^*-/-*^ mice (Fig 6). The *Mtl5*^*c-mu/c-mu*^ mice showed smaller testes than their *Mtl5*^*+/+*^ littermates, with a significant decrease in the testis/body weight ratio to 2.16, which is comparable to that of *Mtl5*^*-/-*^ mice (Fig 6A and 6B). H&E staining showed spermatogenic arrest at the primary spermatocyte stage in *Mtl5*^*c-mu/c-mu*^ mice, which was indistinguishable from that of *Mtl5*^*-/-*^ mice (Fig 6D). In agreement with these findings, no sperm were observed in the cauda epididymis from *Mtl5*^*c-mu/c-mu*^ mice, same as that seen in the *Mtl5*^*-/-*^ mice cauda epididymis (Fig 6C and 6D). This observation is also consistent with those in the reported *Mtl5* knockout mice [21]. Additionally, all of the germ cells were SYCP3 positive, with no detectable PNA positive cells observed in both *Mtl5*^*c-mu/c-mu*^ and *Mtl5*^*-/-*^ seminiferous tubules, further confirming the arrest of spermatogenesis in the primary spermatocyte stage in *Mtl5*^*c-mu/c-mu*^ mice (S8 Fig).

To further determine the exact stage at which meiosis is arrested in *Mtl5*^*c-mu/c-mu*^ mice, we performed immunostaining for SYCP3 and SYCP1, the synapsis makers between homologous chromosomes, on spermatocyte spreads to examine meiotic prophase progression in *Mtl5*^*+/+*^, *Mtl5*^*c-mu/c-mu*^, and *Mtl5*^*-/-*^ mice. It is shown that 207 diplotene spermatocytes, out of 921 meiotic prophase I cells examined, were observed in *Mtl5*^*+/+*^ testes, while no diplotene cells were seen in either *Mtl5*^*c-mu/c-mu*^ or *Mtl5*^*-/-*^ testes after scoring 767 and 976 prophase I spermatocytes, respectively (Fig 6E and 6F). Compared with those in controls, the ratios of leptotene and zygotene spermatocytes increased significantly in *Mtl5*^*c-mu/c-mu*^ and *Mtl5*^*-/-*^ testes due to the lack of diplotene spermatocytes, while the ratio of pachytene spermatocytes was not significantly changed, indicating loss of pachytene spermatocytes in *Mtl5*^*c-mu/c-mu*^ and *Mtl5*^*-/-*^ mice (Fig 6E and 6F). Consistent with this, TUNEL assay showed a significant increase in the number of apoptotic spermatocytes in testes of *Mtl5*^*c-mu/c-mu*^ and *Mtl5*^*-/-*^ mice (S9 Fig).

Given the similar ratios of spermatocytes at different sub-stages in *Mtl5*^*c-mu/c-mu*^ and *Mtl5*^*-/-*^ mice, we further determined whether the C-terminal mutation of MTL5 cause spermatogenesis arrest at the same stage with *Mtl5*^*-/-*^ mice. First, we compared the testicular histomorphological sections at 10, 12, 15, 20, and 25 days post-partum (dpp) from control, *Mtl5*^*c-mu/c-mu*^, and *Mtl5*^*-/-*^ mice, and observed nuclear morphology abnormality in germ cells from *Mtl5*^*c-mu/c-mu*^ and *Mtl5*^*-/-*^ mice since 15 dpp, which were distinguishable from control mice, indicating spermatogenesis arrests between 12 to 15 dpp (before late pachytene) in both *Mtl5*^*c-mu/c-mu*^ and *Mtl5*^*-/-*^ mice (S10 Fig). To further confirm the spermatogenesis arrest before late pachytene stage, we stained H1t on *Mtl5*^*+/+*^, *Mtl5*^*c-mu/c-mu*^, and *Mtl5*^*-/-*^ spermatocyte spreads (S11 Fig). We found no pachytene spermatocytes with thicker ends of lateral elements or strong H1t signal (characteristics of late pachytene) in *Mtl5*^*c-mu/c-mu*^ and *Mtl5*^*-/-*^ mice, indicating similar spermatogenesis arrest before late pachytene in both mutant mice (S11 Fig).

To examine whether homologous recombination was affected in *Mtl5*^*c-mu/c-mu*^ and *Mtl5*^*-/-*^ mice, we counted foci of meiotic DSB markers RPA2 (S12A and S12B Fig) and DMC1 (S12C and S12D Fig) in zygotene and early-pachytene spermatocytes. No obvious difference in the numbers of RPA2 or DMC1 foci were observed in *Mtl5*^*c-mu/c-mu*^ zygotene and early-pachytene spermatocytes, when compared with the wild-type controls (S12 Fig). We also stained SYCP1 and SYCP3 to detect synapsis of pachytene spermatocytes in *Mtl5*^*+/+*^, *Mtl5*^*c-mu/c-mu*^, and *Mtl5*^*-/-*^ mice. We found that no synapsis defects were observed in about 60% pachytene spermatocytes, even in the remained pachytene spermatocytes, the synapsis defects were only involved in one or two autosomal pairs in the mutants (S13 Fig). Thus, it indicates that there is only a mild synapsis defect in our mutant mice and this synapsis defect is not likely the main cause of pachytene arrest.

**Fig 7:**
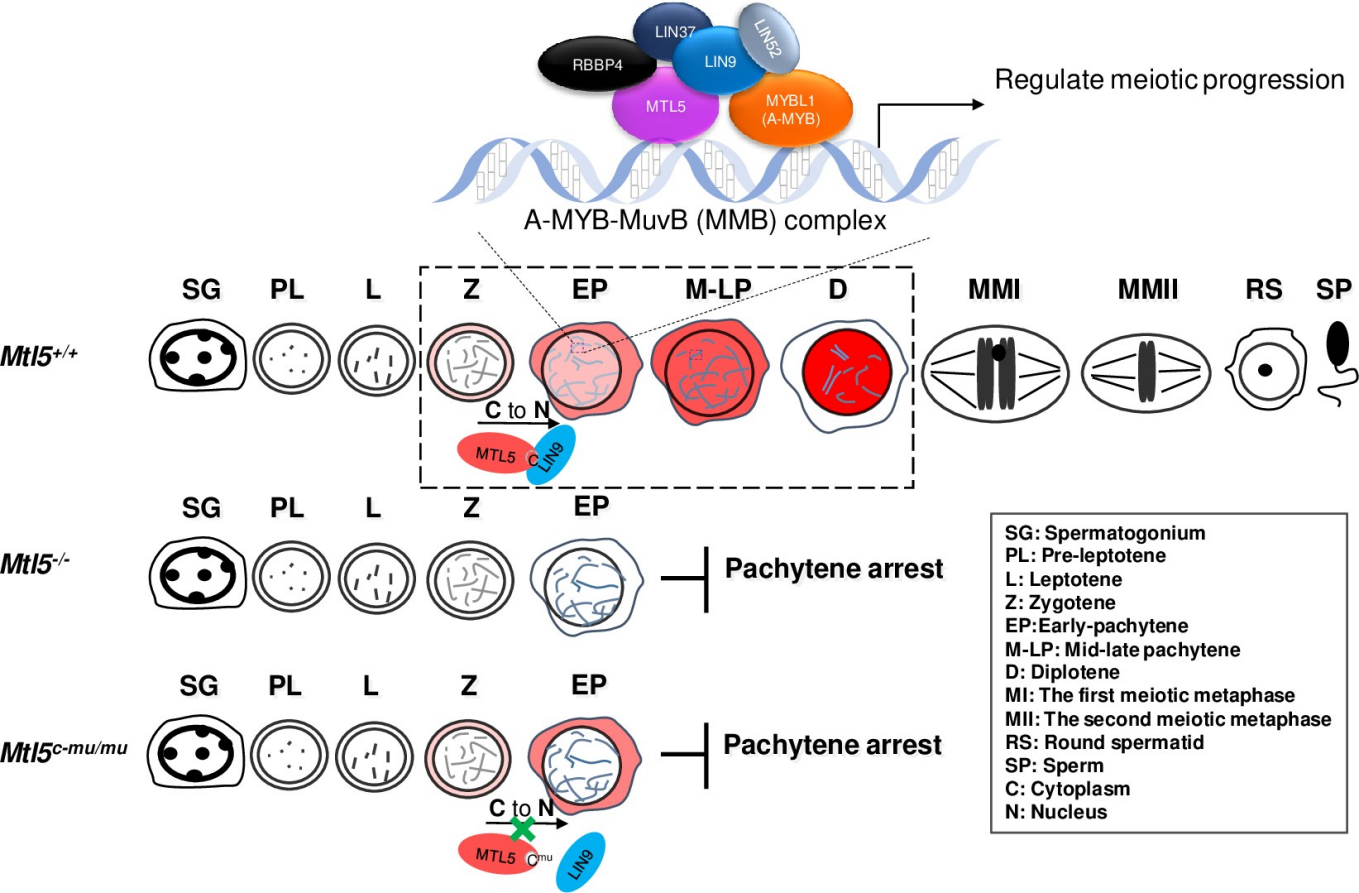
A predicted brief work model of MTL5 in regulating male meiosis. C to N represent MTL5 translocated from cytoplasm (C) to nucleus (N).

Taken together, these results indicate that the nuclear translocation of MTL5 in spermatocytes, mediated via direct interaction with LIN9, is essential for meiotic progression during spermatogenesis, sperm production, and thus for male fertility (Fig 7).

## Discussion

In the current study, we characterized the dynamic localization of MTL5 in spermatocytes of different meiotic sub-stages and highlighted the significance of MTL5 nuclear translocation in the zygotene-pachytene transition during spermatogenesis. We showed that the MTL5 protein accumulates in the cytoplasm of leptotene and zygotene spermatocytes and then begins to translocate into the nuclei of spermatocytes around the zygotene-pachytene transition. Further experiments indicated that MTL5, mediated by its C-terminus, interacts directly with LIN9, a component of MuvB core complex, by which it is transported into the nuclei of spermatocytes from the cytoplasm. The reproductive phenotype of MTL5 mutant (deleting C-terminal 445–475 aa of MTL5) and knockout mice demonstrated that the nuclear translocation of MTL5 in the spermatocytes is required for MTL5 to function, as the deletion of MTL5 C-terminal 445–475 aa prevented its nuclear translocation and led to meiosis arrest at the pachytene stage during spermatogenesis, exactly the same phenotype as observed in *Mtl5*^*-/-*^ mice.

Spermatogenesis is a complex developmental progress and requires a series of proteins to shuttle between the cytoplasm and nuclei of germ cells [29]. For example, cyclin D2 was observed in the nuclei of pachytene spermatocytes (G2 stage), and cyclin D3 was detected in the cytoplasm of elongating spermatids [30,31], while in somatic cells, the D-type cyclins are generally accumulated in the nucleus at the G1 phase and then are rapidly exported to cytoplasm for degradation at the onset of the S phase [32,33], suggesting a specific mechanism that regulates the nuclear and cytoplasmic transition of functional proteins. However, it remained unknown how this mechanism is achieved to facilitate spermatogenesis. Here, we identified the localization of MTL5 in spermatocytes and revealed that MTL5 begins to translocate into the nuclei around the zygotene-pachytene transition. Thus, the specific cytoplasm and nucleus distribution of MTL5 in spermatocytes of different stages provides us a novel model to understand the mechanism and function of the cytoplasmic-nucleus translocation of proteins during spermatogenesis.

The translocation of proteins into the nucleus is a fundamental process and is critical to the basic function of cells. Although many proteins move into the nucleus by the conserved nuclear localization sequence (NLS) [34,35], no canonical NLS is observed in the MTL5 sequence. We demonstrated that the MTL5 cytoplasmic-nucleus translocation is achieved by LIN9, which is a nuclear and chromatin-associated protein [36], through their direct interaction. Prevention of the interaction between MTL5 and LIN9 results in cytoplasmic retention of MTL5, which consequently leads to spermatogenesis arrest at the pachytene stage, same as observed in *Mtl5*^*-/-*^ mice. Intriguingly, no LIN9 protein was observed in leptotene and zygotene spermatocytes in wild-type testicular sections (S6A Fig) and LIN9 protein was first detected in the spermatocytes around 12 dpp (S6B Fig), which is consistent with the nucleus translocation of MTL5, further confirming the essential role of LIN9 in transporting MTL5 into the nuclei of spermatocytes around the zygotene-pachytene transition. Thus, the transportation of MTL5 into the nuclei of spermatocytes by LIN9 is necessary for meiosis progress beyond the pachytene stage during spermatogenesis.

*Mtl5* knockout mice was reported to experience arrest of spermatogenesis at the pachytene stages, with approximately 18.8% spermatocytes at the late pachytene and diplotene stages [21]. However, we did not find late pachytene or diplotene spermatocytes in either *Mtl5*^*c-mu/c-mu*^ or *Mtl5*^*-/-*^ mice. This inconsistency may be due to the criteria used to distinguish different meiotic sub-stages [9,37,38]. Oji *et al*. defined the late-pachytene and diplotene spermatocytes based on the formation of an intact sex body, which could also be observed in early or mid-pachytene spermatocytes [39–41]. However, based on the spermatocyte spreads stained for synaptonemal complex (SC) lateral element protein (SYCP3) and central element protein (SYCP1) as well as H1t staining signal, we did not find any cells with strong H1t signal and thicker ends of lateral elements or desynapsed SCs, characteristics of late pachytene or diplotene spermatocytes [37], in *Mtl5*^*c-mu/c-mu*^ or *Mtl5*^*-/-*^ mice, indicating the arrest of spermatogenesis occurred before the late pachytene stage in these mice.

Homologous recombination and synapsis defects are two major causes of meiotic arrest at pachytene stage. However, we detected no obvious difference in the numbers of RPA2 and DMC1 foci among *Mtl5*^*+/+*^, *Mtl5*^*c-mu/c-mu*^ and *Mtl5*^*-/-*^ spermatocytes at zygotene and early-pachytene stage, indicating the meiotic arrest in *Mtl5* mutants was not a result of homologous recombination defect. Though we did observe mild synapsis deficiency in *Mtl5*^*c-mu/c-mu*^ and *Mtl5*^*-/-*^ spermatocytes, the severity of the asynapsis is not expected to result in meiotic pachytene arrest [42,43].

We have demonstrated that MTL5 interacts with MYBL1 and all MuvB core complex components except LIN54 in mouse testes and cultured human somatic cells. This interaction with the MuvB core complex has been demonstrated in *Arabidopsis thaliana* [44]. It has been shown recently that TCX5, a tesmin/TSO1-like CXC domain-containing protein, is a component of the TCX5/6-containing multi-subunit complexes, which are also known as DREAM complex (a complex containing E2F/DP-p130/p107 and MuvB core complex) [44,16,17].The MuvB core complex is known to play essential roles during various phases of cell cycle, by binding and directing different key transcription factors to the promoters of cell cycle genes [26,45,28,16,17]. During mitosis, the MuvB core complex binds E2F/DP-p130/p107 to form the DREAM complex to repress cell cycle dependent gene expression in the G0 and early G1 phases, while it recruits B-MYB (MYBL2) proteins to form the Myb-MuvB (MMB) complex during the S and early G2 phases and recruits FOXM1 to the promoters of the G2/M genes to activate their expression [46,26,28,17]. In meiosis, deletion of MYBL1 (A-MYB, the paralog of B-MYB), which is highly expressed after meiotic entry, also leads to spermatogenic arrest before the late pachytene stage [47], the similar meiotic arrest stage in MTL5 deficiency mice, indicating an essential role for the Myb-MuvB complex in promoting meiotic progression through the mid-pachytene stage. Given that MYBL1 is pulled down by MTL5 together with the MuvB core complex components in the testes, our findings indicates that MTL5 is essential for meiosis progression beyond the mid-pachytene stage during spermatogenesis and that its deficiency leads arrest of spermatogenesis at the pachytene and consequently to azoospermia. Thus, it is likely that MTL5, after its nuclear translocation mediated by binding to LIN9 of the MuvB core complex, functions together with the Myb-MuvB complex to promote meiosis progression beyond the mid-pachytene stage during spermatogenesis (Fig 7).

It is well-known that cyclins and cyclin-dependent kinases (CDKs) are well-defined regulators of the cell cycle. In meiosis, cyclin/CDK complex is known to have unique characteristics and requirements [48,49,11,50–54,13]. Some of them (such as *Ccnb1*, *Ccnd2* and *Ccnb2*) only affect the meiotic cell cycle in female mice. Furthermore, deletion of Cyclin A1, which is highly expressed in testis, leads to male meiotic arrest at mid-diplotene [48,55];Cyclin E1 deficient testes are morphologically normal as wild-type testes, Cyclin E2 deletion leads to spermatid maturation arrest [50], while double mutant of Cyclin E1 and E2 causes meiotic arrest at a pachytene-like stage with severe synapsis defects [50];CDK2 is essential for the attachment of telomere to the nuclear envelope, and its deficiency leads to defects in homolog pairing due to the failure of bouquet formation [51]. Given the unique phenotype caused by *Mtl5* mutation, we think that MTL5 regulates meiotic progression by affecting factors which are previously unknown.

MTL5 can pull down all of the components of the MuvB core complex except LIN54, a paralog of MTL5 [18,56] and is expressed at low levels in mouse testes (S3 Fig). In somatic cells, LIN54 was reported to serve as a key factor for directing the MuvB core complex to bind the cell cycle genes homology region (CHR), an regulatory element in the promoter regions of cell cycle-regulated genes[18], through its DNA binding domain (CRC domain) which shares sequence similarity with MTL5[18,57,56]. Thus, we believe that MTL5 substitutes the function of LIN54 in the MuvB core complex and then directs the MuvB core complex to the promoters of essential genes required in pachytene spermatocytes. Taken together, our results showed that MTL5 is translocated into the nucleus by LIN9 to promote meiotic progression beyond the pachytene stage. The function of MTL5 is likely achieved by substituting LIN54 to form a meiosis-specific MuvB core complex to promote the meiotic cell cycle progression. Thus, our study not only identified MTL5 as a novel and germ-cell specific regulator of cell cycle progression to function at a specific stage in meiosis to ensure male fertility, but also sheds light on further research to find the master regulatory proteins that directly promote meiotic progression (**Fig 7**).

## Materials and methods

### Ethics statement

All the experiments on mice followed the guidelines of the Institutional Animal Care Committee of the University of Science and Technology of China with the approval number USTCACUC1301021.

### Mice

CRISPR/Cas9 genome editing was used to generate *Mtl5* knockout and mutant mice as described previously [58,59]. The sgRNAs and Cas9 mRNA were microinjected into the zygotes of C57BL/6J mice, and the zygotes were then transferred into pseudo-pregnant ICR females. Genomic DNA was extracted from toe biopsies of founder mice for Sanger sequencing, and heterozygous founder mice were bred to produce homozygous mice. The founder female mice with a 19 bp deletion in exon 2 or a 76 bp deletion in exon 9 of *Mtl5* (ENSMUST00000025840.15) was selected to produce homozygous *Mtl5* knockout (*Mtl5*^*-/-*^) and mutant (*Mtl5*^*c-mu/c-mu*^) mice, respectively. All the mice were nourished with proper food, ddH_2_O and maintained in a 12-hour photoperiod (lights on 08:00–20:00). The primers for genotyping and sgRNAs used in this study are listed in S2 Table.

### Hematoxylin and eosin (H&E) staining and immunohistochemistry

The mice were euthanized by cervical dislocation, testes and epididymides were detached and immediately fixed overnight in Bouin’s solution for H&E staining or in 4% paraformaldehyde (PFA) for immunohistochemistry at 4°C. For immunohistochemistry, testicular and epidydimal sections were incubated with the primary antibodies overnight at 4°C, and then probed with secondary antibodies for 1 hour at 37°C mounting with Vectashield (Vector Laboratories, H-1000) containing Hoechst 33342 (Invitrogen, H21492). To reduce inter-experiment variation, testes from wild-type, knockout, and mutant mice were processed simultaneously. Slides were visualized and all images were captured using a Nikon Eclipse 80i microscope equipped with digital cameras (Nikon DS-Ri1 for H&E staining or Hamamatsu C4742-80 for immunohistochemistry). Some images were also captured using a Nikon C2 Plus Confocal Laser Scanning Microscope system.

### RNA isolation and RT-PCR

Total RNA extraction and cDNA synthesis were performed using Trizol reagents (TaKaRa, 9109) and the PrimeScript RT kit (TaKaRa, RR047A) according to the respective manufacturer’s protocols. RT-PCRs were performed using EasyTaq DNA Polymerase (TransGen Biotech, AP111) under the following conditions: 94°C for 3 min, followed by 25~30 cycles of 30 s at 94°C, 60°C for 30 s, and at 72°C for 30 s. The cDNA templates (S1B Fig) from previous studies in our laboratory were used to RT-PCR, as previously described [25]. Primers used for RT-PCR are listed in S2 Table.

### Spermatocyte spreads and immunofluorescence staining

Spermatocyte spreads were prepared and immunofluorescence staining was performed as we previously described [25,60,61]. Vectashield (Vector Laboratories, H-1000) was used to seal the slides and images of meiotic cells were captured using a BX61 microscope (Olympus) connected to a CCD camera, and analyzed using Image-Pro Plus software (Media Cybernetic). The antibodies used for meiotic analysis are listed in S3 Table.

### Co-immunoprecipitation (Co-IP) and mass spectrometry

The pachytene and diplotene spermatocytes were enriched using STA-PUT method [25] and then were lysed in cold RIPA buffer (Beyotime, P0013C) with phenylmethylsulfonyl fluoride (Thermo Fisher, 36978) and protease inhibitor cocktail (Roche, 04693116001). The lysates were sonicated for 8–10 cycles (3 s on/off) with 8% pulses and centrifuged at 15,000 xg in 4°C for 15 minutes. The supernatant was divided into two aliquots, and each aliquot was incubated with pre-cleared Protein A/G agarose beads (Santa Cruz, sc-2003) and 2 μg anti-MTL5 C-terminal antibody (epitope from residues 221–475) or rabbit IgG non-specific antibody (ABclonal, AC005). After overnight incubation at 4°C, the agarose beads were washed in RIPA buffer for 3 times and twice in 10 mM Tris buffer, and then the immunocomplexes were dissociated from the beads with elution buffer (0.2 M Glysine, 0.15% NP-40, pH 2.3) at room temperature. After validation of the eluted samples by silver staining, samples were analyzed by mass spectrometry in National Center for Protein Science Shanghai. The candidate interacting proteins of MTL5 are listed in S1 Table.

### TUNEL assay

Cell apoptosis was detected in testicular sections by terminal deoxynucleotidyl transferase dUTP nick end labeling (TUNEL) assay according to the manufacturer’s specifications (Roche, 11684795910, Basel, Switzerland). The images were captured using a Nikon ECLPSE 80i microscope (Nikon) which is equipped with a CCD camera (Hamamatsu) and analyzed with the NIS-Element Microscope imaging software (Nikon).

### Cytoplasmic and nuclear protein extraction

The extraction of cytoplasmic and nuclear proteins was performed according to the manufacturer’s specifications (Beyotime, P0027). GAPDH and SYCP3 were served as the loading controls of cytoplasm and nucleus, respectively.

### Western blot

Western blot was carried out as we previously described [25] and the bands were visualized and examined with chemiluminescence (GE Healthcare, ImageQuant LAS 4000). The antibodies used in this study are listed in S3 Table.

### Plasmids

The typical mouse *Mtl5* (ENSMUST00000025840.15) full-length coding sequence was reverse transcribed from mouse testis cDNAs and cloned into a MYC-tagged expression vector under control of CMV promotor to form MYC-*Mtl5*. The mouse *Lin9*, *Lin37*, *Lin52*, *Rbpp4*, *Mybl1*, and *Lin54* full-length coding sequences were also reverse transcribed from mouse testis cDNAs, and cloned into the pEGFP-N1 expression vector to form GFP-*Lin9*, GFP-*Lin37*, GFP-*Lin52*, GFP-*Rbbp4*, GFP-*Mybl1*, and GFP-*Lin54* through the ClonExpress II one Step Cloning kit (Vazyme, C113) following the manufacturer’s protocol. The fragments of MTL5-N (1–250 aa), MTL5-M (251–370 aa), MTL5-C1 (371–418 aa), MTL5-C2 (419–442 aa), and MTL5-C3 (443–475 aa) were deleted from MTL5 to construct MYC-*Mtl5*-ΔN, MYC-*Mtl5*-ΔM, MYC-*Mtl5*-ΔC1, MYC-*Mtl5*-ΔC2, and MYC-*Mtl5*-ΔC3, respectively. In addition, the mouse typical *Mtl5* full-length coding sequence was fused to GAL4 activation domain (AD) vector pGADT7 or GAL4 BD/bait vector pGBKT7 to form AD-MTL5 or BD-MTL5 to detect the auto-activation. Mouse *Lin9*, *Lin37*, *Lin52*, *Rbbp4*, and *Mybl1* full-length coding sequences were cloned into GAL4 BD/bait vector pGBKT7 to form BD-LIN9, BD-LIN37, BD-LIN52, BD-RBBP4, and BD-MYBL1. The N (1–250 aa), M (251–370 aa), C (371–475 aa), C1 (371–418 aa), C2 (419–442 aa), and C3 (443–475 aa) fragments of MTL5 were cloned into pGADT7 vector to construct plasmids AD-MTL5-N, AD-MTL5-M, AD-MTL5-C, AD-MTL5-C1, AD-MTL5-C2, and AD-MTL5-C3, respectively. The truncated fragments N (1–150), M (151–340), and C (341–559) of LIN9 were cloned into pGBKT7 vector to construct BD-LIN9-N, BD-LIN9-M, and BD-LIN9-C. All primers used for plasmid construction are listed in S2 Table.

### The transfection, immunofluorescence and Co-IP in cultured cells

HEK293T cells (ATCC, CRL-3216) were cultured in high-glucose Dulbecco’s Modified Eagle’s Medium (DMEM) supplemented with 10% FBS (GIBCO, 15140122) with 100 U/ml penicillin and 100 ug/ml streptomycin (GIBCO, 16000044) and maintained in 5% CO_2_, ambient O_2_ at 37°C. Cells were passaged at least twice after thawing and transfected at 70%-80% cell density. The transfection was performed using lipofectamine 3000 (Invitrogen) according to the manufacturer’s protocol.

Twenty-four hours of after transfection, cells were fixed in 4% paraformaldehyde followed by immunofluorescence. The primary antibodies were incubated at 4°C overnight and followed with secondary antibodies for 1 hr at 37°C. Finally, the cells were mounted with Vectashield. Pictures were captured using a Nikon C2 Plus Confocal Laser Scanning Microscope. The antibodies used for cell immunofluorescence are listed in S3 Table.

For Co-IP, the cells were harvested 36 hours after transfection and lysed in pre-cold NP-40 buffer (Beyotime, P0013F) with phenylmethylsulfonyl fluoride (Thermo Fisher, 36978) and protease inhibitor cocktail (Roche, 04693116001). Lysate was then centrifuged and 1/10 volumes of supernatant were used as input samples. The remaining supernatant was incubated with Myc-Tag (9B11) mouse mAb (Magnetic bead conjugate) (Cell Signaling Technology, #5698). After incubation at 4°C overnight with rotation, the magnetic beads were washed in NP-40 buffer and collected by magnetic stand. The proteins were eluted from the beads with sample buffer on a 100°C hot block for 10 min.

### Yeast two-hybrid

The yeast cells transformed with prey (AD) and bait (BD) strains were hybridized in 2xYPDA medium (2% Yeast extracts, 4% Peptone, 4% D-Glucose, and 0.006% adenine hemisulfate) at 30°C overnight, then the hybridized strains were centrifuged, resuspended in 100 ul ddH_2_O, and diluted to different ratios (10^0^, 10^−1^, 10^−2^ and 10^−3^) before being spread on double (SD-Leu/Trp, -LW), triple (SD-His/Leu/Trp with 5 mM 3-AT, -LWH), or quadruple dropout medium plates (SD-Ade/His/Leu/Trp, -LWHA) at 30°C for 3–5 days. Empty pGBKT7 and pGADT7 were used for testing self-activation. Primers used for yeast plasmid construction are listed in S2 Table.

### Statistical analysis

The testis/body weight ratio, the spermatocyte proportions, TUNEL positive tubules and cells analysis, RPA2 and DMC1 foci analysis, H1t-staining analysis were compared between the wild-type, knockout, or mutant mice using the One-way ANOVA. The percentages of co-transfected cells with different MTL5 localization were analyzed by Student’s *t*-test. The results were presented as means ± SEM. Statistical significance was set at *P* < 0.05.

## Supporting information

S1 Fig The expression of MTL5 in mouse tissues and male germ cells. (A) Western blot analysis of MTL5 protein in different tissues of 10-week-old male mice. (B) Western blot analysis of MTL5 protein in 8, 10, 12, 15, 20 and 25 dpp mouse testes (with high exposure to indicate no band in 8 dpp). β-Actin served as loading control. (C) RT-PCR analysis of *Mtl5* mRNA (ENSMUST00000025840.15) in purified mouse spermatogenic cells. SC, Sertoli cells;PL, pre-leptotene spermatocytes;pLZ, pubertal leptotene and zygotene spermatocytes;pPD, pubertal pachytene and diplotene spermatocytes;aPD, adult pachytene and diplotene spermatocytes;RS, round spermatids.
(TIF)

S2 Fig The expression of *Lin54* in different mouse tissues. RT-PCR analysis of *Mtl5* and *Lin54* expression in different tissues of 10-week-old mice. *Actb* served as the internal reference.
(TIF)

S3 Fig MTL5 directly interacts with LIN9 and RBBP4. The interaction of AD-MTL5 with BD-LIN9, BD-LIN37, BD-LIN52, BD-RBBP4, BD-MYBL1, or empty control (pGBKT7) was assessed by Y2H system, on double (SD-Leu/Trp, -LW), triple (SD-His/Leu/Trp with 5 mM 3-AT, -LWH) or quadruple (SD-Ade/His/Leu/Trp, -LWHA) dropout medium plates with different dilutions.
(TIF)

S4 Fig Plasmids constructed for Y2H and HEK293T cell transfection assay. (A) Full length and truncated MTL5 were cloned into GAL4 activation domain (AD) vector pGADT7 to generate AD-MTL5, AD-MTL5-N (1–250 aa), AD-MTL5-M (251–370 aa), AD-MTL5-C (371–475 aa), AD-MTL5-C1 (371–418 aa), AD-MTL5-C2 (419–442 aa) or AD-MTL5-C3 (443–475 aa). (B) Full length and truncated LIN9 were cloned into GAL4 BD/bait vector pGBKT7 to generate BD-LIN9, BD-LIN9-N (1–150 aa), BD-LIN9-M (151–340 aa) or BD-LIN9-C (341–559 aa). (C) The fragments of MTL5-N (1–250 aa), MTL5-M (251–370 aa), MTL5-C1 (371–418 aa), MTL5-C2 (419–442 aa) or MTL5-C3 (443–475 aa) were deleted from MYC-*Mtl5* to generate MYC-*Mtl5*-ΔN, MYC-*Mtl5*-ΔM, MYC-*Mtl5*-ΔC1, MYC-*Mtl5*-ΔC2, or MYC-*Mtl5*-ΔC3, respectively. The gray boxes represent the cysteine-rich domains (CRC). The green and red boxes represent the regions containing helix domains predicted by SWISS-MODEL. del, deletion;aa, amino acid.
(TIF)

S5 Fig The localization of truncated MTL5 proteins in cultured cells. HEK293T cells were transiently transfected with plasmids encoding (A) Myc-*Mtl5-*ΔN, (B) Myc-*Mtl5*-ΔM, (C) Myc-*Mtl5*-ΔC1, and (D) Myc-*Mtl5*-ΔC2 alone, or together with GFP-*Lin9* plasmids. MTL5 and LIN9 proteins were detected by immunostaining with antibodies against MTL5 (red) and GFP (green) and the protein localization was analyzed by a confocal laser scanning microscope. Nuclei (blue) were counterstained with Hoechst 33342. The “n” in brackets indicates the number of co-transfected cells analyzed. Data are presented as mean ± SEM for three independent experiments. **p<0.01;***p<0.001;****p<0.0001. P values were determined by Student’s *t-test*. Scale bars, 10 μm.
(TIF)

S6 Fig The localization and expression of LIN9 in mouse testes. (A) Immunostaining of testicular sections for LIN9 (red) and γH2AX (green) from 10-week-old wild-type mice. Nuclei were counterstained with Hoechst 33342 (blue). Red arrowhead indicates Sertoli cell. Green arrowheads indicate spermatogonia. White arrowheads indicate leptotene or zygotene spermatocytes;Yellow arrowheads indicate pachytene or diplotene spermatocytes. Scale bar, 50 μm. (B) Western blot analysis of LIN9 protein in the tetraploid germ cells (primary spermatocytes) of 8 dpp, 10 dpp, 12 dpp, 14 dpp, 20 dpp and adult (10-week-old) mouse testes. 4C DNA content represent the tetraploid spermatocytes enriched by Fluorescence activated Cell Sorting (FACS).
(TIF)

S7 Fig The generation of Mtl5 knockout (*Mtl5*^*-/-*^) mice. (A) Diagram (top) and nucleotide sequence (bottom) of mouse *Mtl5* locus taken from the Ensemble database (ENSMUST00000025840.15) with exons (green boxes) and TGA (red) premature stop codon of the modified transcript. CF and CR represent the genotyping primers of *Mtl5* knockout mice. PAM, protospacer adjacent motif. sgRNA, single-guide RNA. A mouse strain with a 19 bp deletion starting from position 498 to 516 in exon 2 was confirmed by PCR (B) and Sanger sequencing (C). (D) Western blot analysis of MTL5 expression in testes of 10-week-old mice with corresponding genotypes.
(TIF)

S8 Fig *Mtl5*^*c-mu/c-mu*^ mice fails to generate spermatids and sperm. Immunostaining of testicular sections for PNA (red) and SYCP3 (green) from 10-week-old *Mtl5*^*+/+*^, *Mtl5*^*c-mu/c-mu*^, and *Mtl5*^*-/-*^ mice. Nuclei were counterstained with Hoechst 33342 (grey). The cells in the white dotted boxes are enlarged aside. Scale bars, 50 μm.
(TIF)

S9 Fig Increased apoptosis in *Mtl5*^*c-mu/c-mu*^ and *Mtl5*^*-/-*^ mouse testes. (A) TUNEL assay on *Mtl5*^*+/+*^, *Mtl5*^*c-mu/c-mu*^ and *Mtl5*^*-/-*^ mouse testicular sections. Red, TUNEL positive cells. Scale bars, 50 μm. (B-C) The percentage of tubules with TUNEL positive cells and the number of TUNEL positive cells per tubules in *Mtl5*^*+/+*^, *Mtl5*^*c-mu/c-mu*^, and *Mtl5*^*-/-*^ testes. Numbers in the brackets indicate the number of counted tubules. Data are presented as mean ± SEM. P values were analyzed by One-way ANOVA. ****p<0.0001;NS, p>0.05.
(TIF)

S10 Fig The spermatogenesis in postnatal CONTROL, *Mtl5*^*c-mu/c-mu*^ and *Mtl5*^*-/-*^ mice. H-E staining on testicular sections from control, *Mtl5*^*c-mu/c-mu*^ and *Mtl5*^*-/-*^ mice at indicated age. Red arrows indicated spermatocytes. Yellow arrows indicate metaphase spermatocytes. Blue arrows indicate round spermatids. Black arrows indicated spermatocytes with highly condensed chromatin (apoptotic cells). Scale bars, 50 μm.
(TIF)

S11 Fig The spermatogenesis arrest before late pachytene stage in *Mtl5*^*c-mu/c-mu*^ and *Mtl5*^*-/-*^ mice. (A) Immunostaining of SYCP3 (red), H1t (green), and γH2AX (blue) on pachytene spermatocytes from 10-week-old *Mtl5*^*+/+*^, *Mtl5*^*c-mu/c-mu*^ and *Mtl5*^*-/-*^ mice. N, H1t-negative;P, H1t-positive. (B) The percentages of H1t-negative pachytene spermatocytes in 10-week-old *Mtl5*^*+/+*^, *Mtl5*^*c-mu/c-mu*^ and *Mtl5*^*-/-*^ mice. Numbers in the brackets indicate the number of counted cells. Data are presented as mean ± SEM. P values were analyzed by One-way ANOVA. ****p<0.0001;NS, p>0.05. Scale bars, 10 μm.
(TIF)

S12 Fig Meiotic recombination was not affected in *Mtl5*^*c-mu/c-mu*^ and *Mtl5*^*-/-*^ mice. Immunostaining and quantification of RPA2 (A, B) and DMC1 (C, D) in 10-week-old *Mtl5*^*+/+*^, *Mtl5*^*c-mu/c-mu*^ and *Mtl5*^*-/-*^ zygotene and early-pachytene spermatocytes. Data are presented as mean ± SEM. P values were analyzed by One-way ANOVA. NS, p>0.05. Scale bars, 10 μm.
(TIF)

S13 Fig Mild synapsis defects in *Mtl5*^*c-mu/c-mu*^ and *Mtl5*^*-/-*^ pachytene spermatocytes. (A) Immunostaining of SCYP1 (red) and SYCP3 (green) in *Mtl5*^*+/+*^, *Mtl5*^*c-mu/c-mu*^ and *Mtl5*^*-/-*^ pachytene spermatocytes. White dotted ovals indicate the asynapsis of autosomal pairs. Scale bars, 10 μm. (B) Percentages of cells with defective autosomal synapsed bivalents. Number of cells analysed in brackets.
(TIF)

S1 Table 27 candidate proteins (unique peptides >2) interact with MTL5 identified by mass spectrometry. (DOCX)

S2 Table Primers used in this study. (DOCX)

S3 Table Antibodies used in this study. (DOCX)
