# Supplementary figures and images for: Nuclear translocation of MTL5 from cytoplasm requires its direct interaction with LIN9 and is essential for male meiosis and fertility
Source: PLoS Genet. 2021 Aug 13;17(8):e1009753. doi: 10.1371/journal.pgen.1009753 (PMC8386835; doi:10.1371/journal.pgen.1009753)

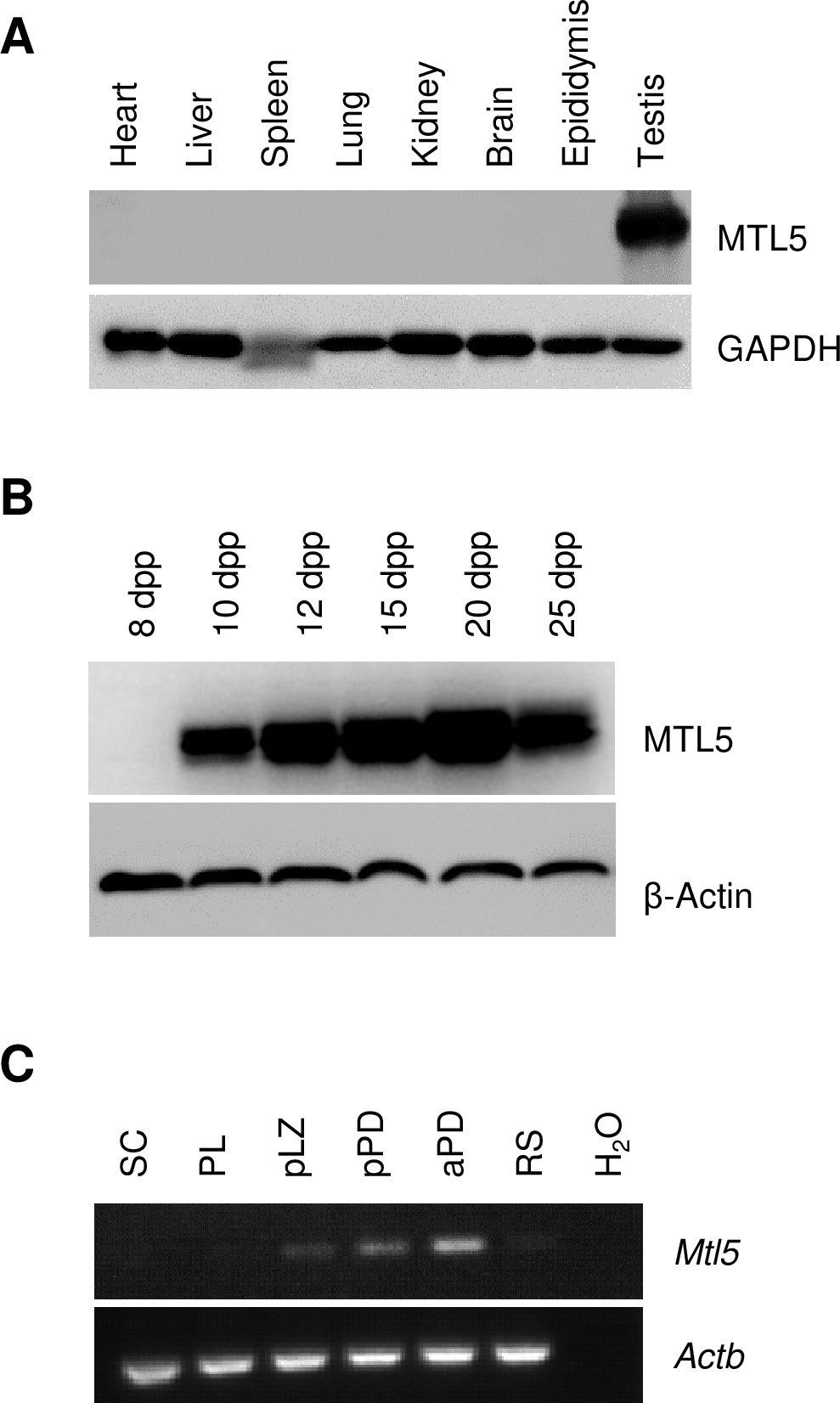

Supplement: S1 Fig — (A) Western blot analysis of MTL5 protein in different tissues of 10-week-old male mice. (B) Western blot analysis of MTL5 protein in 8, 10, 12, 15, 20 and 25 dpp mouse testes (with high exposure to indicate no band in 8 dpp). β-Actin served as loading control. (C) RT-PCR analysis of Mtl5 mRNA (ENSMUST00000025840.15) in purified mouse spermatogenic cells. SC, Sertoli cells;PL, pre-leptotene spermatocytes;pLZ, pubertal leptotene and zygotene spermatocytes;pPD, pubertal pachytene and diplotene spermatocytes;aPD, adult pachytene and diplotene spermatocytes;RS, round spermatids. (TIF) [file pgen.1009753.s001.tif]

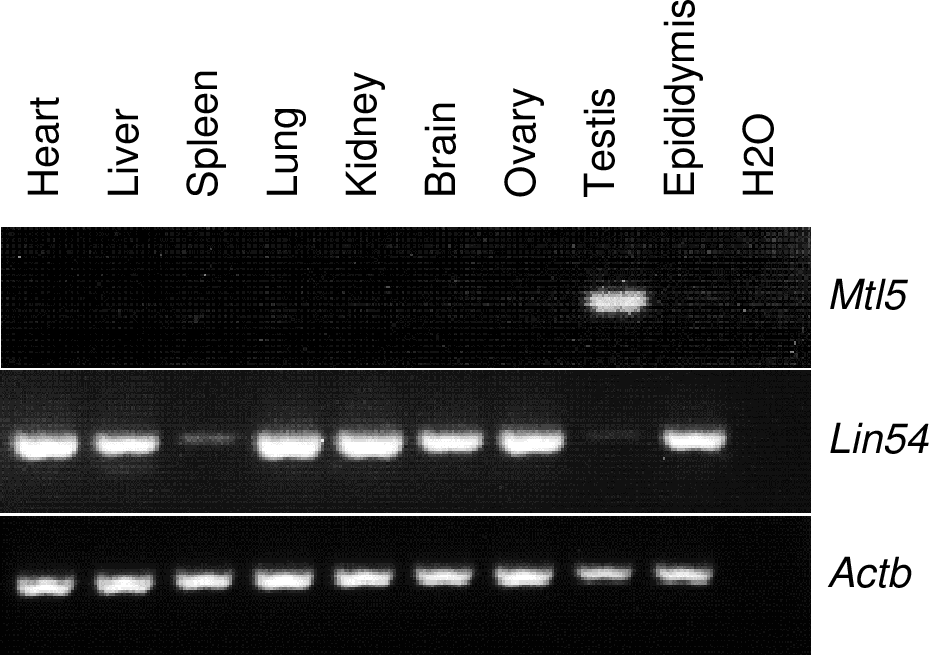

Supplement: S2 Fig — RT-PCR analysis of Mtl5 and Lin54 expression in different tissues of 10-week-old mice. Actb served as the internal reference. (TIF) [file pgen.1009753.s002.tif]

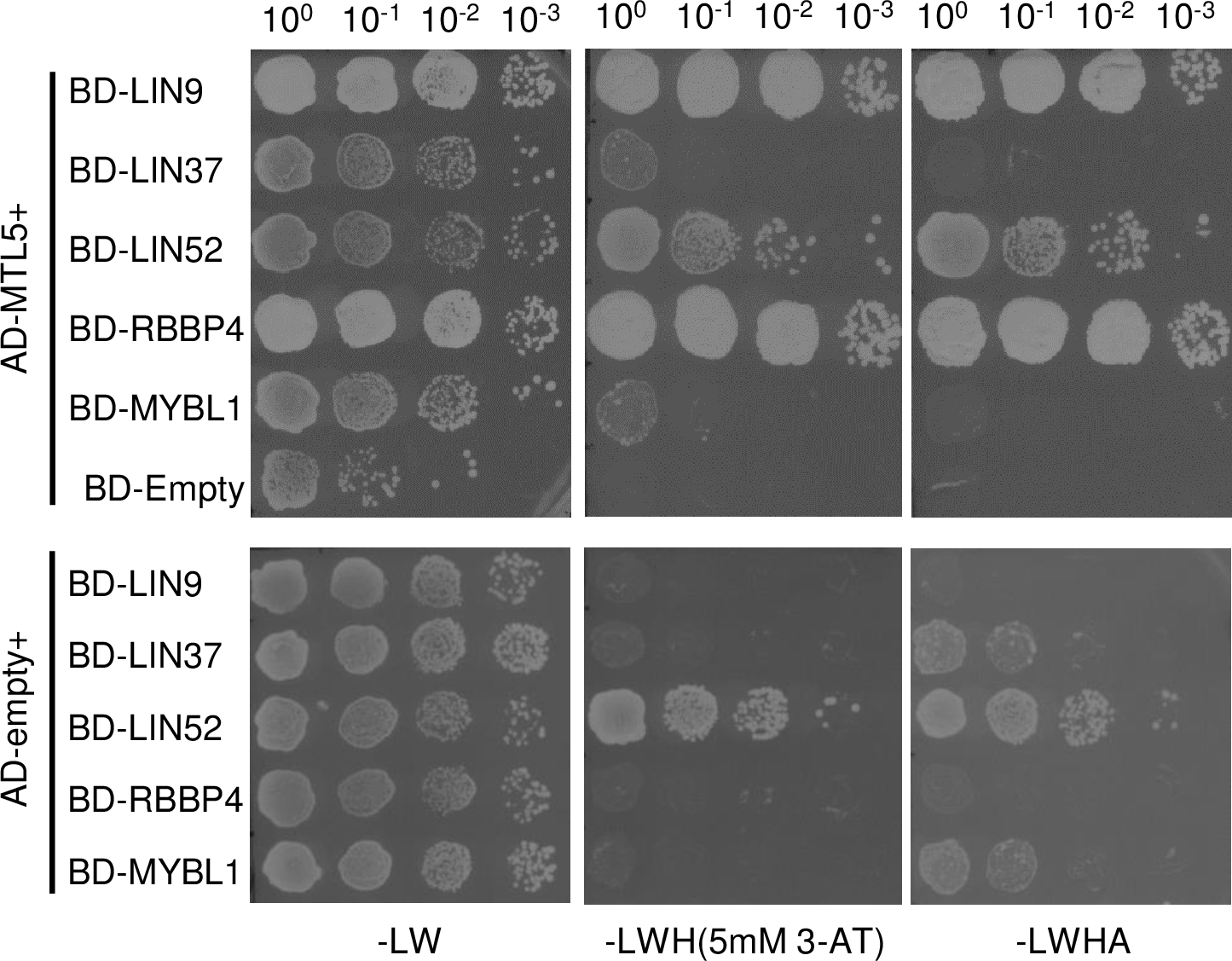

Supplement: S3 Fig — The interaction of AD-MTL5 with BD-LIN9, BD-LIN37, BD-LIN52, BD-RBBP4, BD-MYBL1, or empty control (pGBKT7) was assessed by Y2H system, on double (SD-Leu/Trp, -LW), triple (SD-His/Leu/Trp with 5 mM 3-AT, -LWH) or quadruple (SD-Ade/His/Leu/Trp, -LWHA) dropout medium plates with different dilutions. (TIF) [file pgen.1009753.s003.tif]

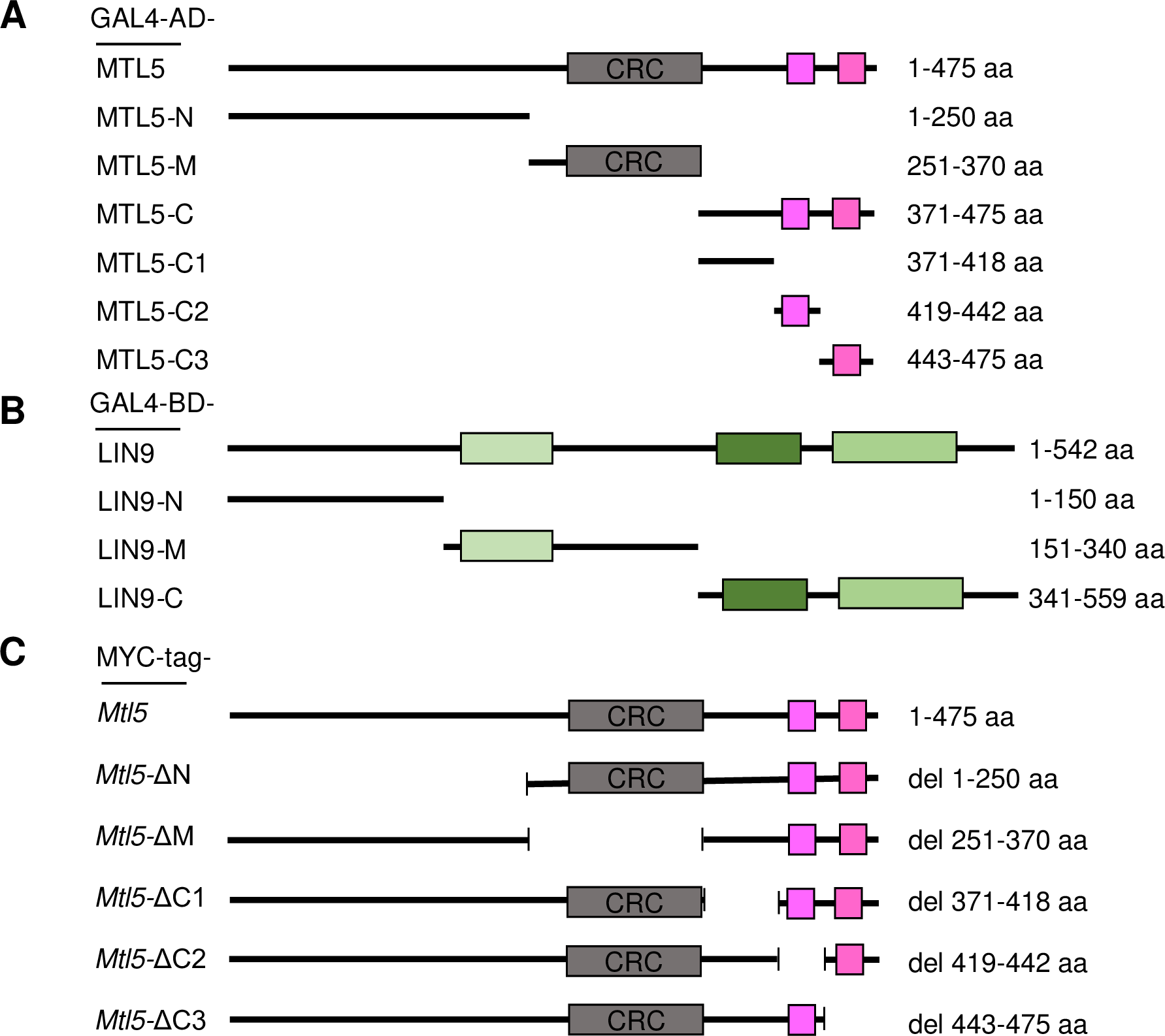

Supplement: S4 Fig — (A) Full length and truncated MTL5 were cloned into GAL4 activation domain (AD) vector pGADT7 to generate AD-MTL5, AD-MTL5-N (1–250 aa), AD-MTL5-M (251–370 aa), AD-MTL5-C (371–475 aa), AD-MTL5-C1 (371–418 aa), AD-MTL5-C2 (419–442 aa) or AD-MTL5-C3 (443–475 aa). (B) Full length and truncated LIN9 were cloned into GAL4 BD/bait vector pGBKT7 to generate BD-LIN9, BD-LIN9-N (1–150 aa), BD-LIN9-M (151–340 aa) or BD-LIN9-C (341–559 aa). (C) The fragments of MTL5-N (1–250 aa), MTL5-M (251–370 aa), MTL5-C1 (371–418 aa), MTL5-C2 (419–442 aa) or MTL5-C3 (443–475 aa) were deleted from MYC-Mtl5 to generate MYC-Mtl5-ΔN, MYC-Mtl5-ΔM, MYC-Mtl5-ΔC1, MYC-Mtl5-ΔC2, or MYC-Mtl5-ΔC3, respectively. The gray boxes represent the cysteine-rich domains (CRC). The green and red boxes represent the regions containing helix domains predicted by SWISS-MODEL. del, deletion;aa, amino acid. (TIF) [file pgen.1009753.s004.tif]

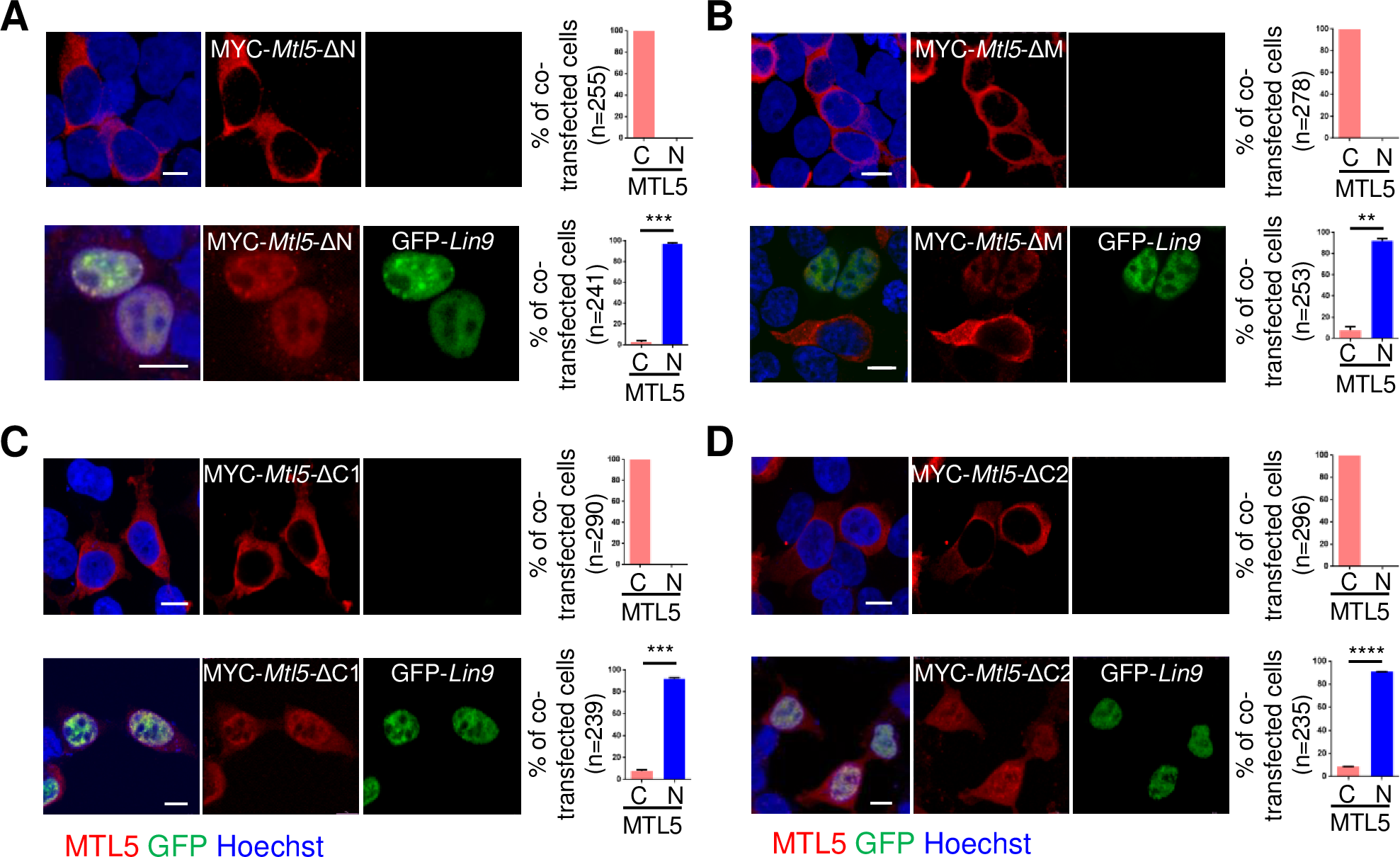

Supplement: S5 Fig — HEK293T cells were transiently transfected with plasmids encoding (A) Myc-Mtl5-ΔN, (B) Myc-Mtl5-ΔM, (C) Myc-Mtl5-ΔC1, and (D) Myc-Mtl5-ΔC2 alone, or together with GFP-Lin9 plasmids. MTL5 and LIN9 proteins were detected by immunostaining with antibodies against MTL5 (red) and GFP (green) and the protein localization was analyzed by a confocal laser scanning microscope. Nuclei (blue) were counterstained with Hoechst 33342. The “n” in brackets indicates the number of co-transfected cells analyzed. Data are presented as mean ± SEM for three independent experiments. **p<0.01;***p<0.001;****p<0.0001. P values were determined by Student’s t-test. Scale bars, 10 μm. (TIF) [file pgen.1009753.s005.tif]

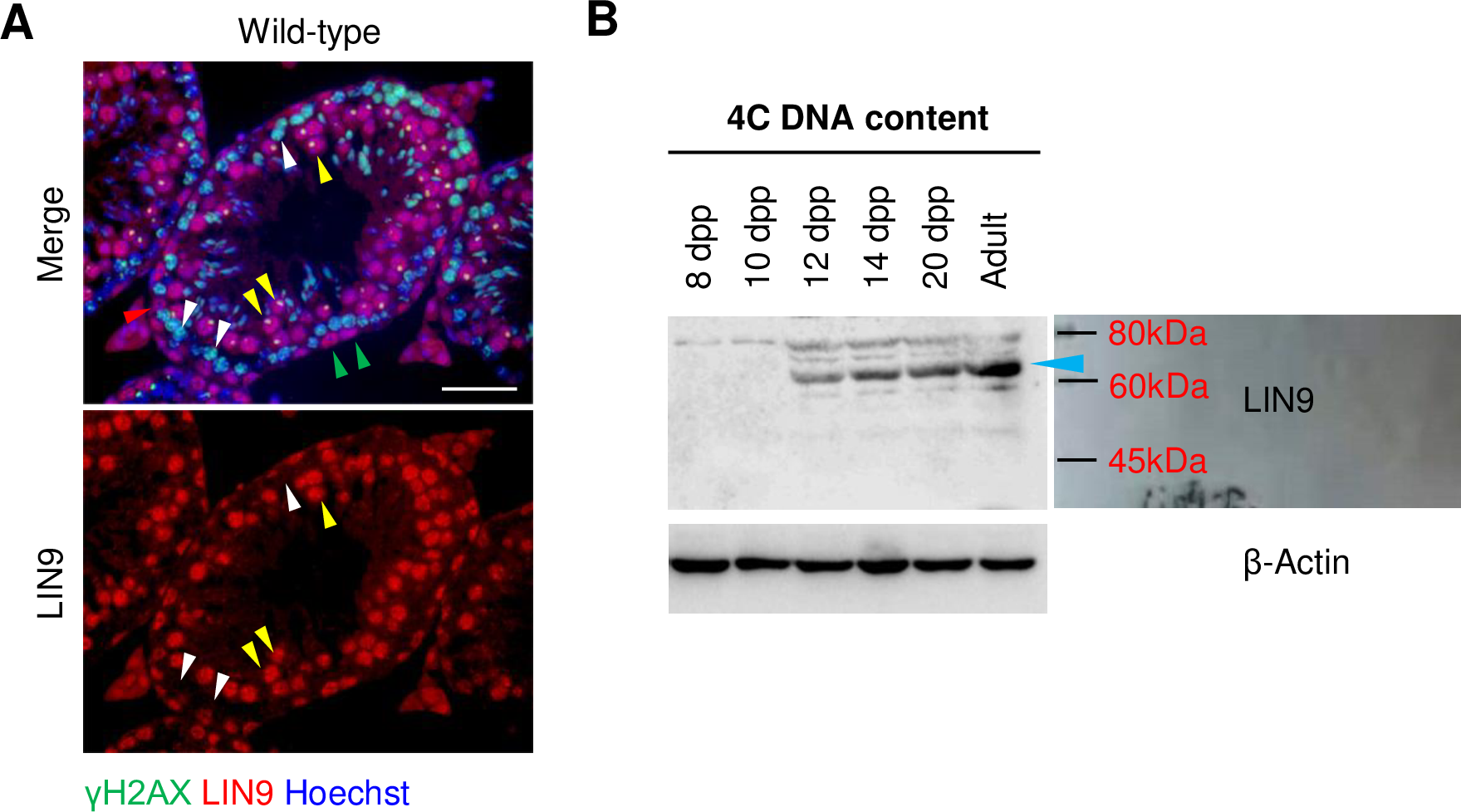

Supplement: S6 Fig — (A) Immunostaining of testicular sections for LIN9 (red) and γH2AX (green) from 10-week-old wild-type mice. Nuclei were counterstained with Hoechst 33342 (blue). Red arrowhead indicates Sertoli cell. Green arrowheads indicate spermatogonia. White arrowheads indicate leptotene or zygotene spermatocytes;Yellow arrowheads indicate pachytene or diplotene spermatocytes. Scale bar, 50 μm. (B) Western blot analysis of LIN9 protein in the tetraploid germ cells (primary spermatocytes) of 8 dpp, 10 dpp, 12 dpp, 14 dpp, 20 dpp and adult (10-week-old) mouse testes. 4C DNA content represent the tetraploid spermatocytes enriched by Fluorescence activated Cell Sorting (FACS). (TIF) [file pgen.1009753.s006.tif]

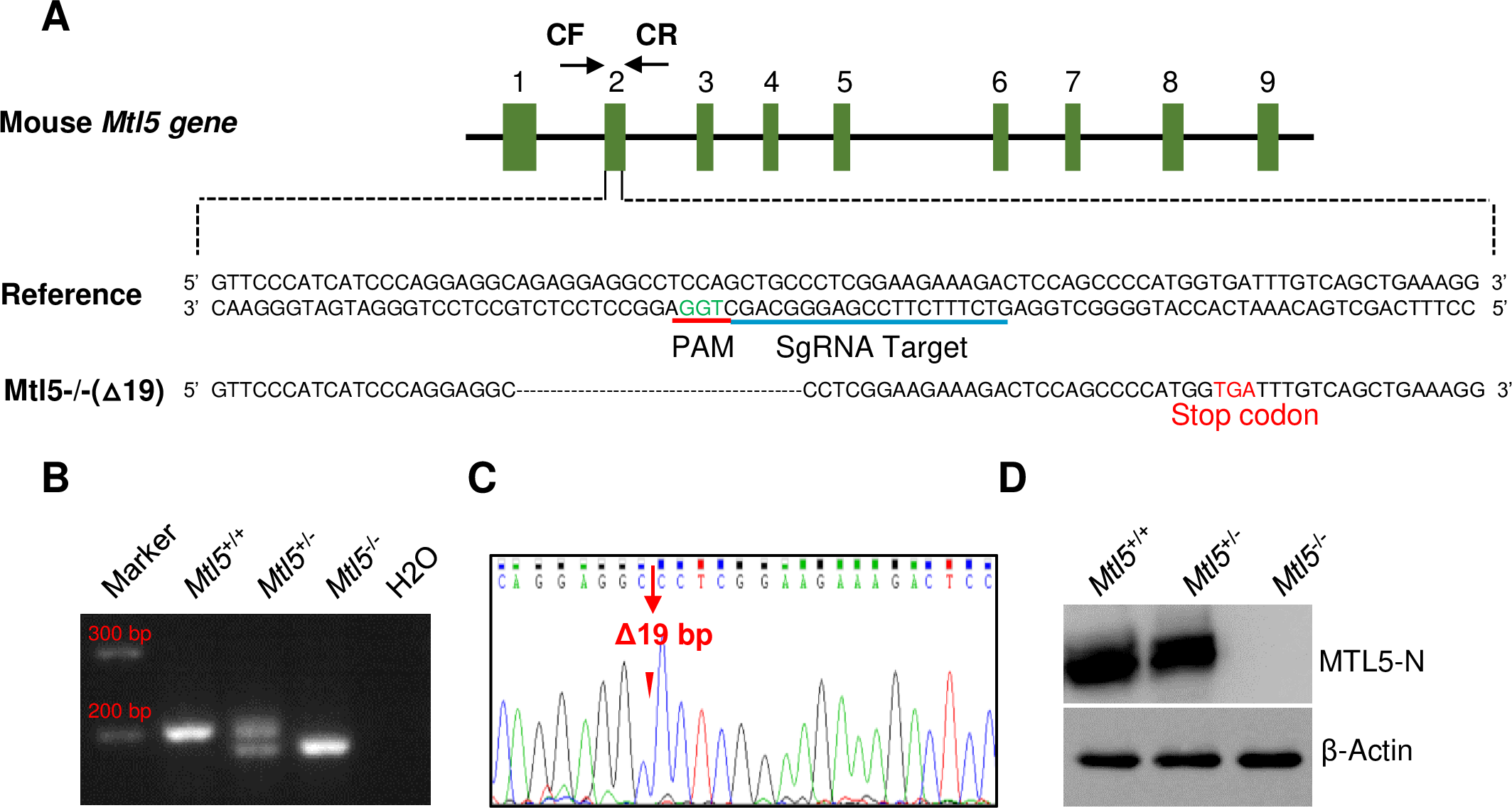

Supplement: S7 Fig — (A) Diagram (top) and nucleotide sequence (bottom) of mouse Mtl5 locus taken from the Ensemble database (ENSMUST00000025840.15) with exons (green boxes) and TGA (red) premature stop codon of the modified transcript. CF and CR represent the genotyping primers of Mtl5 knockout mice. PAM, protospacer adjacent motif. sgRNA, single-guide RNA. A mouse strain with a 19 bp deletion starting from position 498 to 516 in exon 2 was confirmed by PCR (B) and Sanger sequencing (C). (D) Western blot analysis of MTL5 expression in testes of 10-week-old mice with corresponding genotypes. (TIF) [file pgen.1009753.s007.tif]

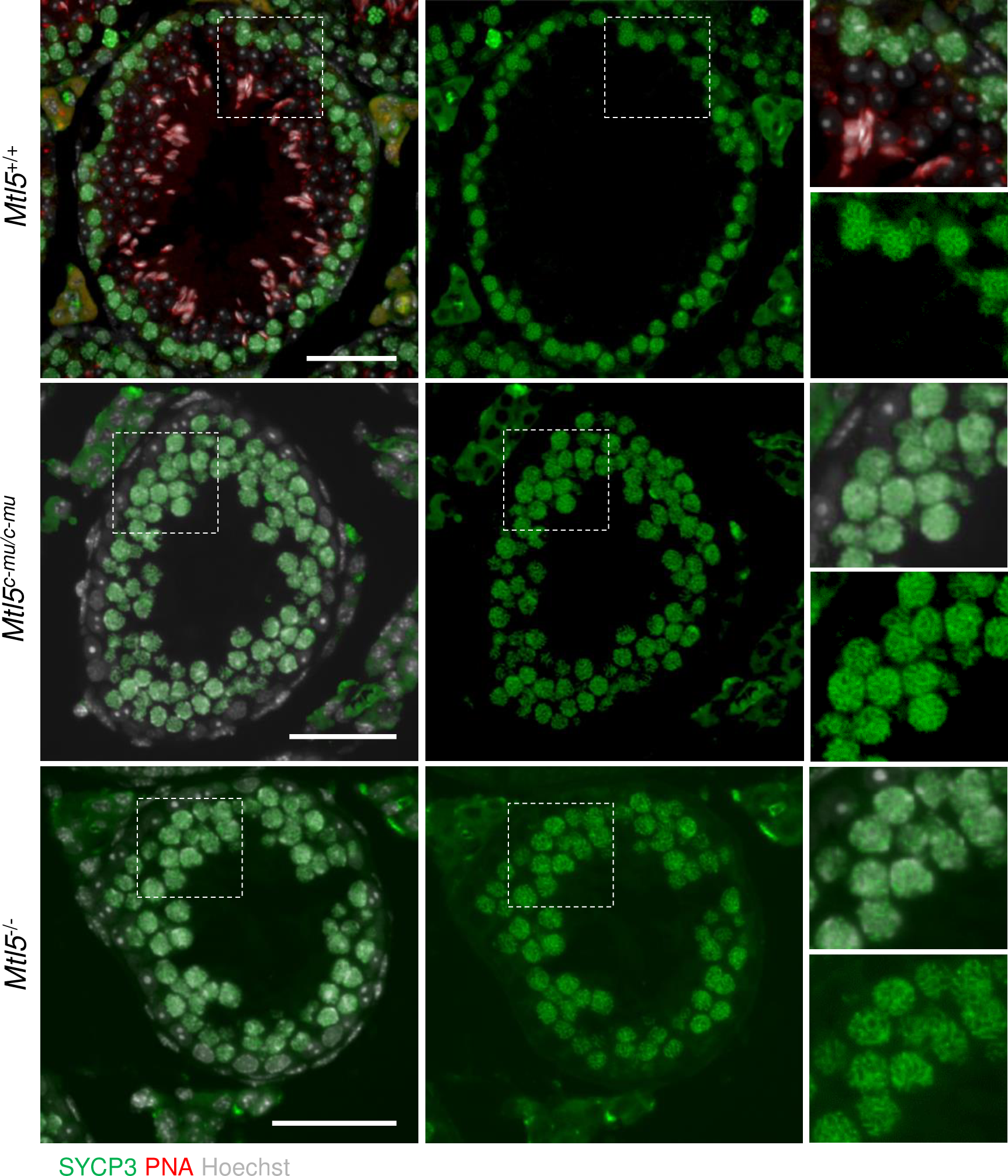

Supplement: S8 Fig — Immunostaining of testicular sections for PNA (red) and SYCP3 (green) from 10-week-old Mtl5+/+, Mtl5c-mu/c-mu, and Mtl5-/- mice. Nuclei were counterstained with Hoechst 33342 (grey). The cells in the white dotted boxes are enlarged aside. Scale bars, 50 μm. (TIF) [file pgen.1009753.s008.tif]

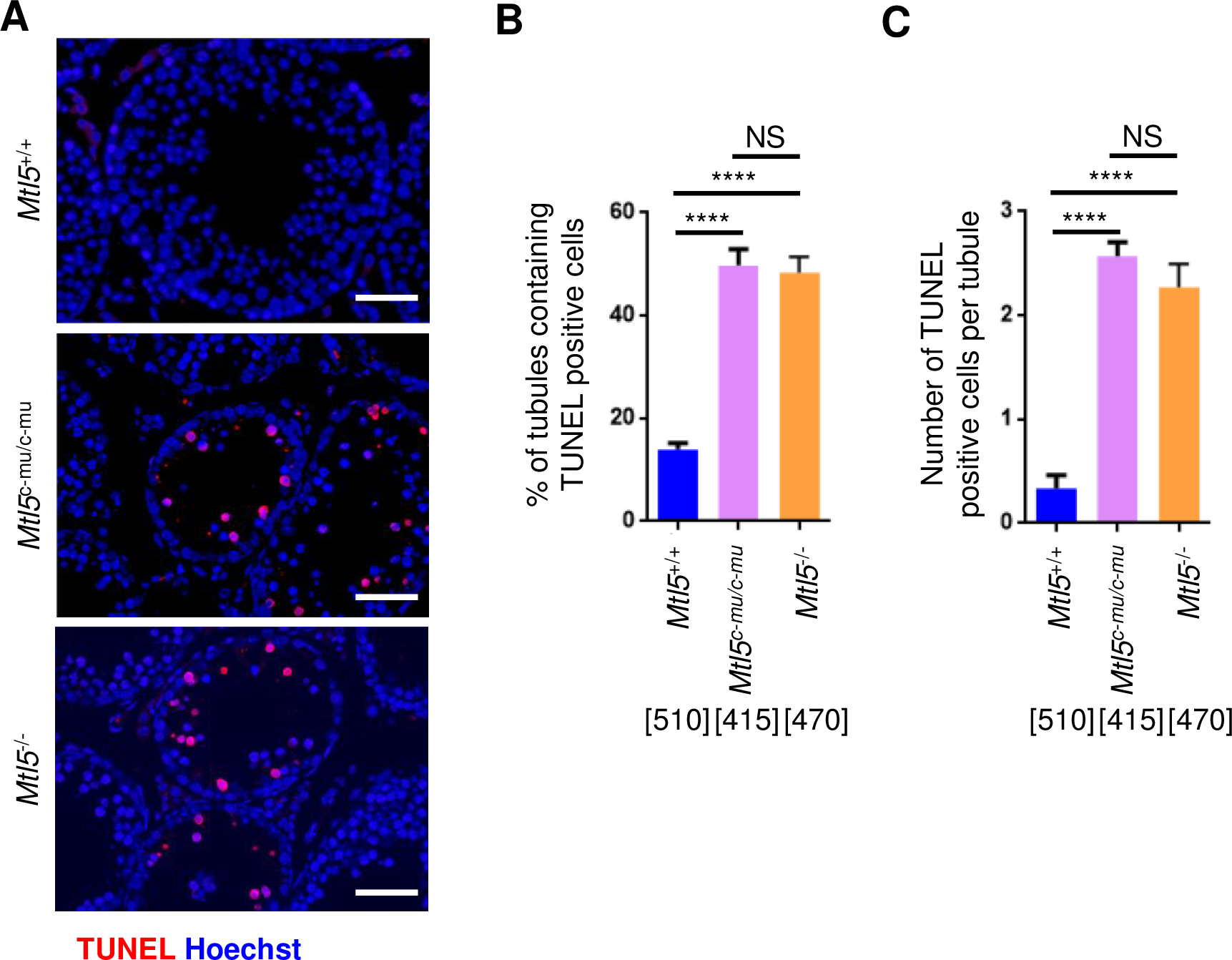

Supplement: S9 Fig — (A) TUNEL assay on Mtl5+/+, Mtl5c-mu/c-mu and Mtl5-/- mouse testicular sections. Red, TUNEL positive cells. Scale bars, 50 μm. (B-C) The percentage of tubules with TUNEL positive cells and the number of TUNEL positive cells per tubules in Mtl5+/+, Mtl5c-mu/c-mu, and Mtl5-/- testes. Numbers in the brackets indicate the number of counted tubules. Data are presented as mean ± SEM. P values were analyzed by One-way ANOVA. ****p<0.0001;NS, p>0.05. (TIF) [file pgen.1009753.s009.tif]

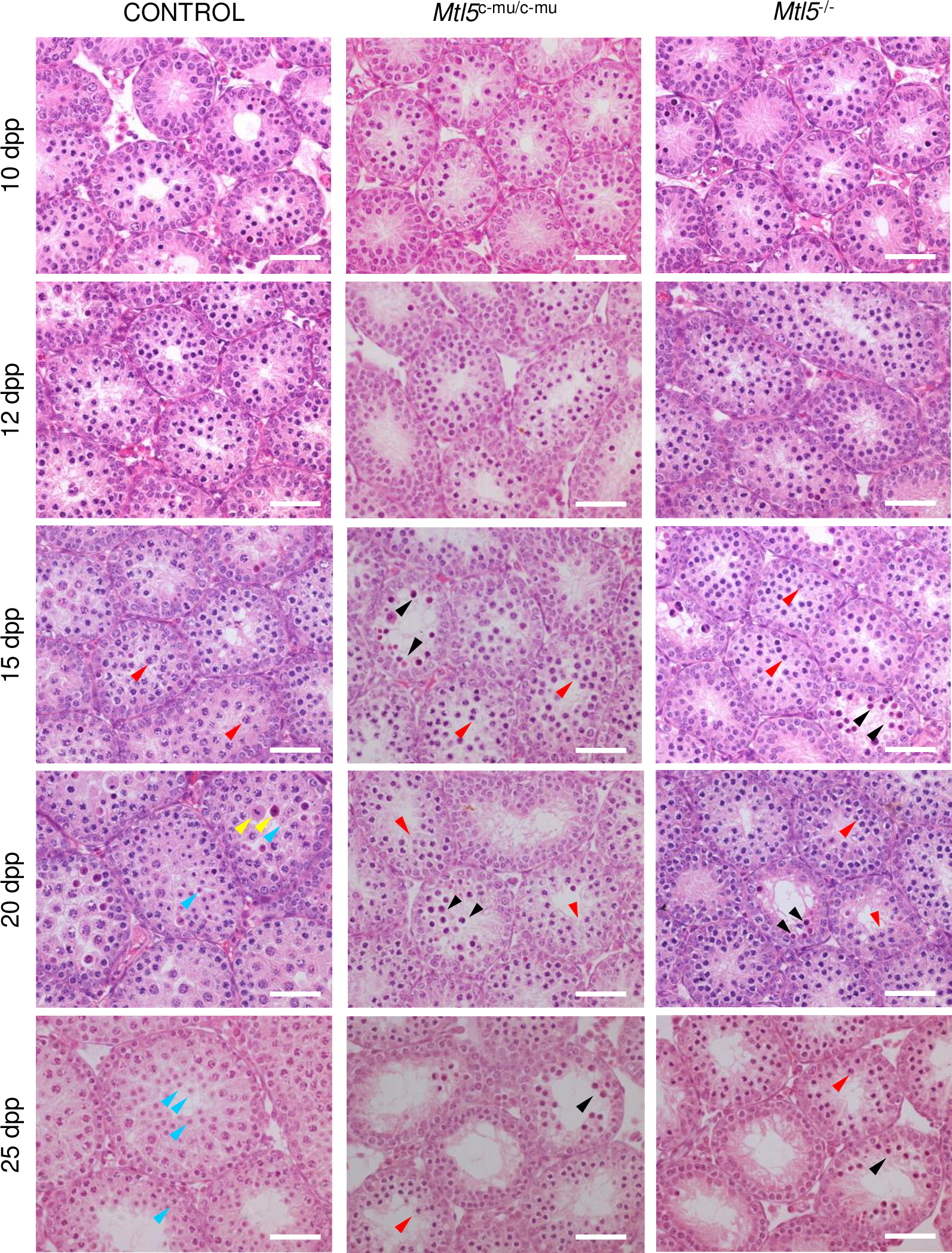

Supplement: S10 Fig — H-E staining on testicular sections from control, Mtl5c-mu/c-mu and Mtl5-/- mice at indicated age. Red arrows indicated spermatocytes. Yellow arrows indicate metaphase spermatocytes. Blue arrows indicate round spermatids. Black arrows indicated spermatocytes with highly condensed chromatin (apoptotic cells). Scale bars, 50 μm. (TIF) [file pgen.1009753.s010.tif]

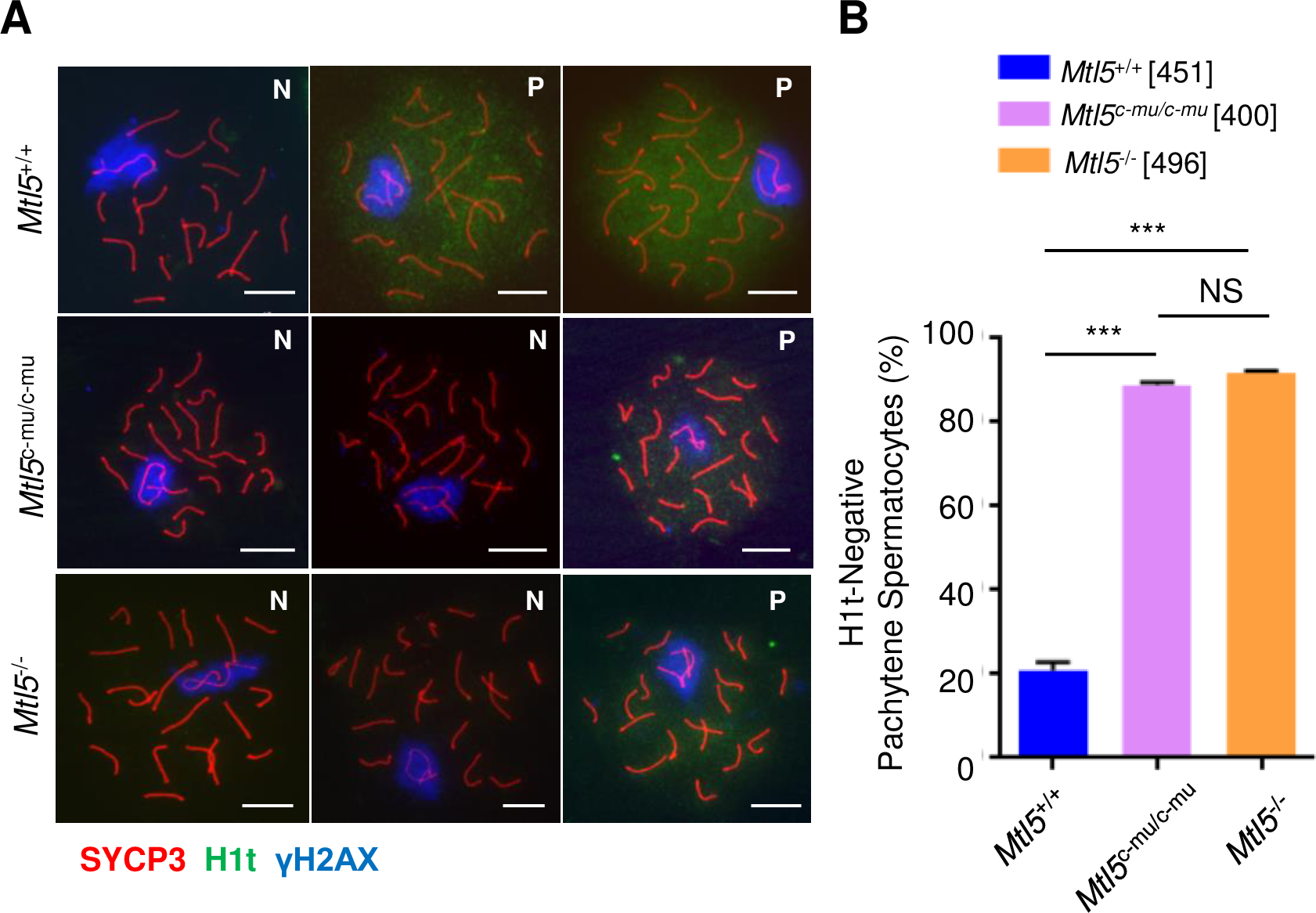

Supplement: S11 Fig — (A) Immunostaining of SYCP3 (red), H1t (green), and γH2AX (blue) on pachytene spermatocytes from 10-week-old Mtl5+/+, Mtl5c-mu/c-mu and Mtl5-/- mice. N, H1t-negative;P, H1t-positive. (B) The percentages of H1t-negative pachytene spermatocytes in 10-week-old Mtl5+/+, Mtl5c-mu/c-mu and Mtl5-/- mice. Numbers in the brackets indicate the number of counted cells. Data are presented as mean ± SEM. P values were analyzed by One-way ANOVA. ****p<0.0001;NS, p>0.05. Scale bars, 10 μm. (TIF) [file pgen.1009753.s011.tif]

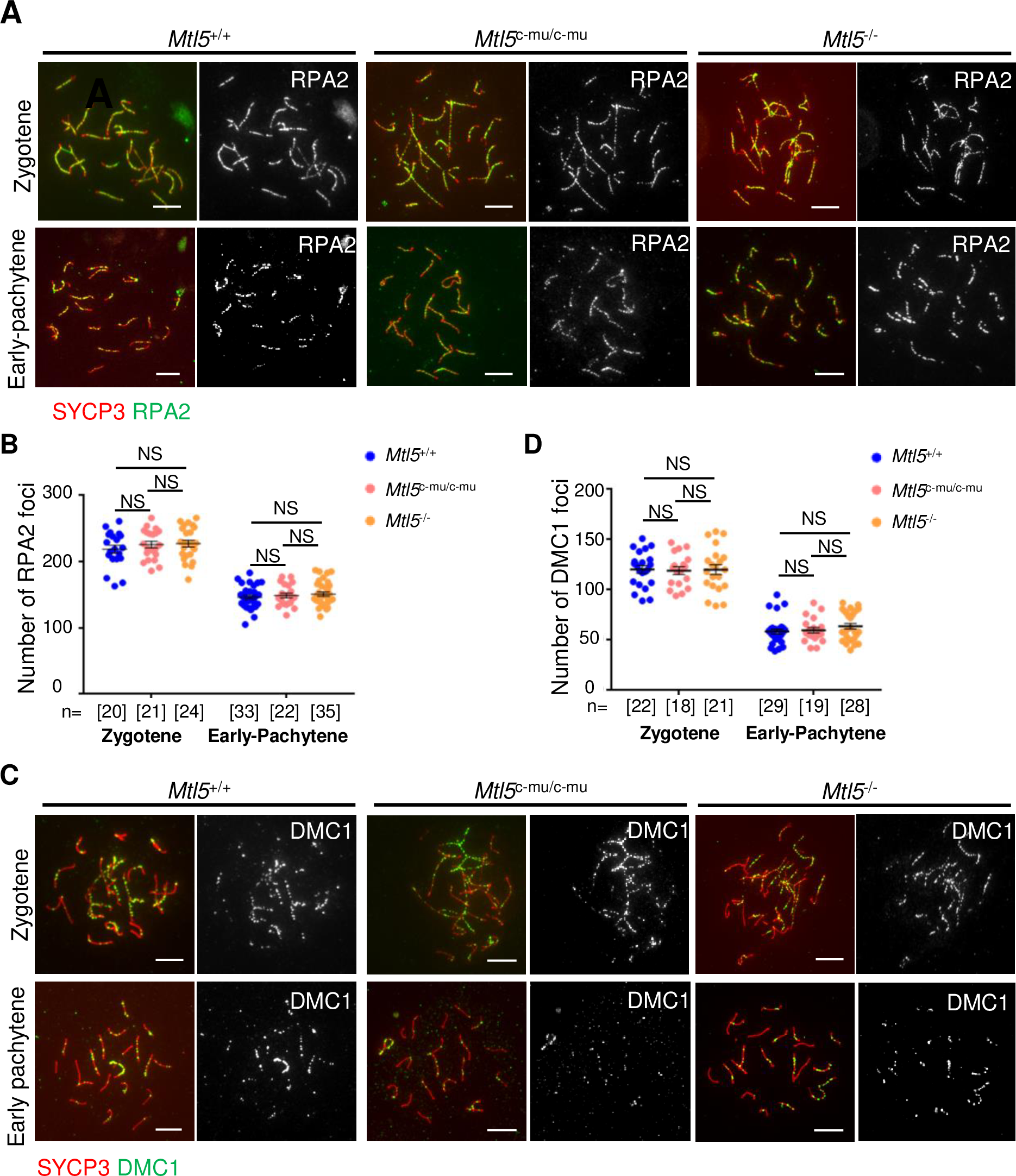

Supplement: S12 Fig — Immunostaining and quantification of RPA2 (A, B) and DMC1 (C, D) in 10-week-old Mtl5+/+, Mtl5c-mu/c-mu and Mtl5-/- zygotene and early-pachytene spermatocytes. Data are presented as mean ± SEM. P values were analyzed by One-way ANOVA. NS, p>0.05. Scale bars, 10 μm. (TIF) [file pgen.1009753.s012.tif]

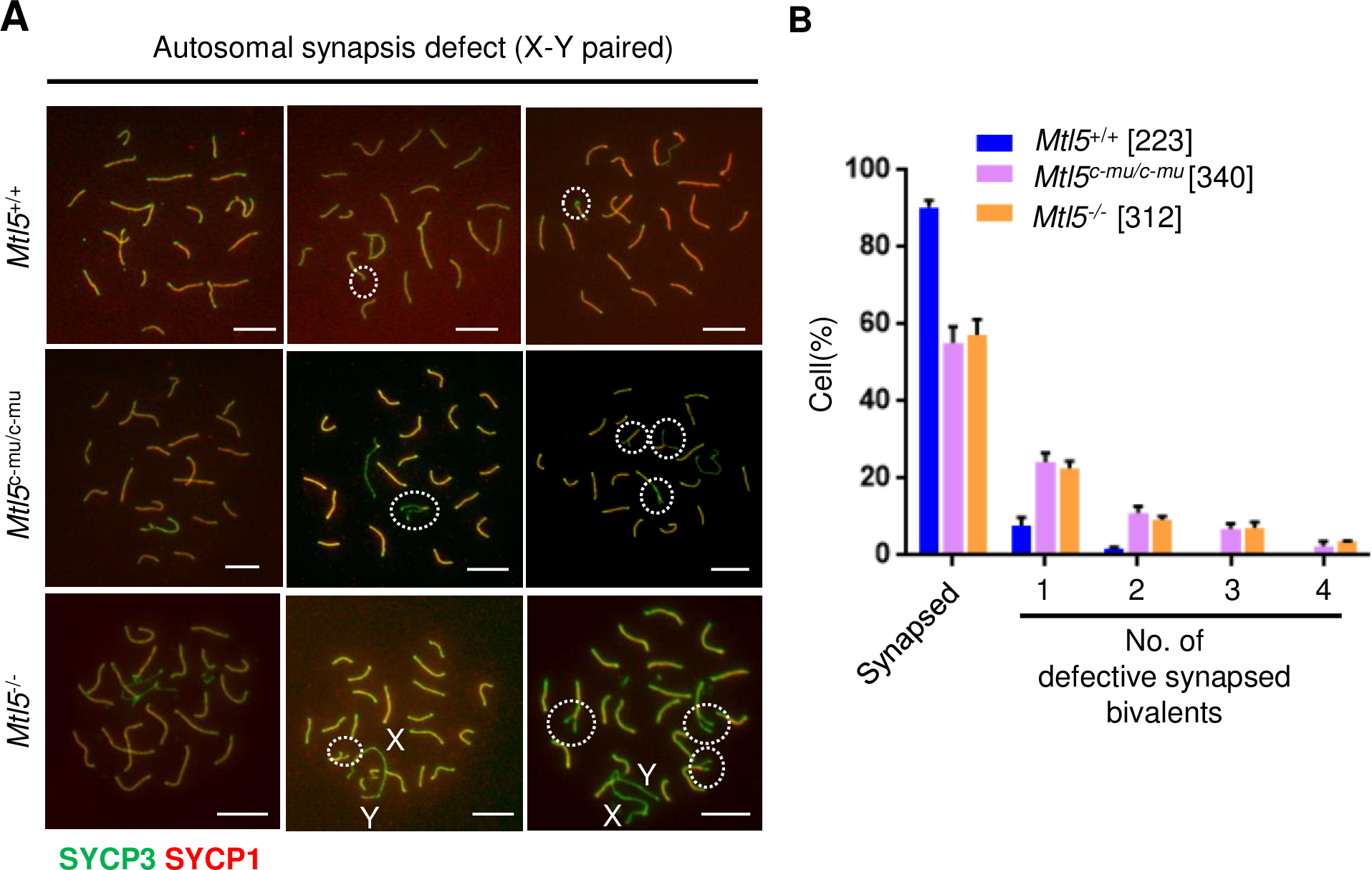

Supplement: S13 Fig — (A) Immunostaining of SCYP1 (red) and SYCP3 (green) in Mtl5+/+, Mtl5c-mu/c-mu and Mtl5-/- pachytene spermatocytes. White dotted ovals indicate the asynapsis of autosomal pairs. Scale bars, 10 μm. (B) Percentages of cells with defective autosomal synapsed bivalents. Number of cells analysed in brackets. (TIF) [file pgen.1009753.s013.tif]
