# Supplementary material for: Nuclear translocation of MTL5 from cytoplasm requires its direct interaction with LIN9 and is essential for male meiosis and fertility
Source: PLoS Genet. 2021 Aug 13;17(8):e1009753. doi: 10.1371/journal.pgen.1009753 (PMC8386835; doi:10.1371/journal.pgen.1009753)
Supplement: S1 Table — (DOCX) [file pgen.1009753.s014.docx]

| **Table S1. 27 candidate proteins (unique peptides >2) interact with MTL5 identified by mass spectrometry** | | | | | | |
| --- | --- | --- | --- | --- | --- | --- |
| **Protein** | **Accession** | **Coverage (%)** | **Unique peptides** | **Length (aa)** | **Description** | **Knockout mice phenotype** |
| **MTL5 (Tesmin)** | **Q9WTJ6** | **82.95** | **21** | **475** | **Testis-specific metallothionein-like protein** | **Meiotic arrest** |
| **RBBP4** | **Q60972** | **54.82** | **12** | **425** | **Nucleosome-remodeling factor subunit RBAP48** | **Embryonic lethality** |
| **LIN9** | **A0A0A6YVZ7** | **46.6** | **23** | **558** | **Component of MuvB core complex** | **Embryonic lethality** |
| **MYBL1** | **Q3ZB50** | **41.51** | **26** | **754** | **Master regulator of male meiosis** | **Meiotic arrest** |
| **LIN52** | **Q8CD94** | **37.93** | **4** | **116** | **Component of MuvB core complex** | **No reference** |
| **LIN37** | **Q9D8N6** | **33.74** | **6** | **246** | **Component of MuvB core complex** | **No reference** |
| **DNAJA4** | **Q9JMC3** | **32.99** | **9** | **397** | **DnaJ heat shock protein family (Hsp40) member A4** | **Fertile** |
| **HSP90AA1** | **Q3UIF3** | **22.92** | **10** | **733** | **Heat shock protein 90, alpha (cytosolic), class A member** | **Meiotic arrest** |
| **1700102P08Rik** | **A0A0R4J0D7** | **15.48** | **3** | **252** | **RIKEN cDNA 1700102P08 gene** | **Meiotic arrest** |
| **NPM1** | **Q3U536** | **13.7** | **3** | **292** | **Nucleophosmin** | **Embryonic lethality** |
| **STK31** | **Q99MW1** | **10.61** | **8** | **1018** | **Serine threonine kinase** | **Fertile** |
| **CEP72** | **Q9D3R3** | **9.44** | **4** | **646** | **Centrosomal protein** | **No reference** |
| **ERLIN2** | **Q8BFZ9** | **8.53** | **3** | **340** | **Endoplasmic reticulum lipid raft-associated protein** | **Fertile** |
| **SETX** | **A2AKX3** | **8.28** | **14** | **2646** | **Probable RNA/DNA helicase** | **Meiotic arrest** |
| **FYCO1** | **Q8VDC1** | **7.17** | **6** | **1437** | **May mediate microtubule plus end-directed vesicle transport** | **No reference** |
| **MEIOC** | **A2AG06** | **6.42** | **4** | **965** | **Meiosis specific with coiled-coil domain** | **Meiotic arrest** |
| **ZMYM4** | **A2A791** | **6.13** | **6** | **1549** | **Cell morphology regulation and cytoskeletal organization** | **No reference** |
| **DDX3X** | **Q3TQX5** | **5.59** | **3** | **662** | **Multifunctional ATP-dependent RNA helicase** | **Fertile** |
| **TDRD1** | **Q99MV1** | **5.55** | **5** | **1172** | **Component of the meiotic nuage** | **Spermatid arrest** |
| **DNAH8** | **Q91XQ0** | **5.16** | **20** | **4731** | **Component of the outer dynein arms (ODAs) in the sperm flagellum** | **No reference** |
| **SYCP1** | **Q62209** | **4.63** | **3** | **993** | **Major component of the transverse filaments of synaptonemal complexes** | **Meiotic arrest** |
| **TDRD6** | **E9PZ50** | **4.36** | **4** | **2135** | **Present in chromatoid body (CB) of spermatids** | **Spermatid arrest** |
| **AKAP3** | **O88987** | **3.7** | **3** | **864** | **May function as a regulator of both motility- and head-associated functions in sperm** | **No reference** |
| **YTHDC2** | **B2RR83** | **2.84** | **4** | **1445** | **3'-5' RNA helicase, promoting transition from mitotic to meiotic divisions in stem cells** | **Meiotic arrest** |
| **TDRD9** | **Q14BI7** | **2.82** | **3** | **1383** | **Component of the nuage, repressing transposon activity during meiosis** | **Meiotic arrest** |
| **MOV10L1** | **D3YWG8** | **2.5** | **3** | **1239** | **Required to repress transposon activity during meiosis** | **Meiotic arrest** |
| **USP34** | **F6WJB7** | **2.05** | **4** | **3602** | **Ubiquitin carboxyl-terminal hydrolase** | **Fertile** |
