# Supplementary material for: Nuclear translocation of MTL5 from cytoplasm requires its direct interaction with LIN9 and is essential for male meiosis and fertility
Source: PLoS Genet. 2021 Aug 13;17(8):e1009753. doi: 10.1371/journal.pgen.1009753 (PMC8386835; doi:10.1371/journal.pgen.1009753)
Supplement: S2 Table — (DOCX) [file pgen.1009753.s015.docx]

| **Table S2. Primers used in this study** | | |
| --- | --- | --- |
| **Primers** | **Sequence (5’-3’)** | **Product size (bp)** |
| *Mtl5*-sgRNA-Top | TAGGGCTGCCCTCGGAAGAAAGAC |  |
| *Mtl5*-sgRNA-Bottom | AAACGTCTTTCTTCCGAGGGCAGC |  |
| *Mtl5*-C^mu^-sgRNA 1-Top | TAGGAAGCTTGGCTGAGCAGATGA |  |
| *Mtl5*-C^mu^-sgRNA 1-Bottom | AAACTCATCTGCTCAGCCAAGCTT |  |
| *Mtl5*-C^mu^-sgRNA 2-Top | TAGGCACATCGAGTTCAAGTCCAA |  |
| *Mtl5*-C^mu^-sg RNA 2-Bottom | AAACTTGGACTTGAACTCGATGTG |  |
| *Mtl5*-check-Fw | AAGAAGCAGGTGGTAGTGTG | *Mtl5^+/+^*:202  *Mtl5^-/-^* : 183 |
| *Mtl5*-check-Rv | CTTGGACTGGCTGAGTGATG |  |
| *Mtl5*-C^mu^-Fw | GACAACTAGAGTGATTGGGT | *Mtl5^+/+^*: 380  *Mtl5^c-mu/c-mu^* : 304 |
| *Mtl5*-C^mu^-Rv | TACCATGCAGATCACTCAGA |  |
| *Mtl5*-RT-Fw | ATCGTCGTTACTTCCCGGTG | 226 |
| *Mtl5*-RT-Rv | GGGGCTGGAGTCTTTCTTCC |  |
| *Lin54*-RT-Fw | TACCAGTCGGCCCAGAAAAC | 493 |
| *Lin54*-RT-Rv | TCCTGCACTGTTGAGAGCTG |  |
| Mouse *Actb*-Fw | ACCAACTGGGACGACATGGAGAA | 214 |
| Mouse *Actb*-Rv | TACGACCAGAGGCATACAGGGAC |  |
| MYC-*Mtl5*-Fw | CATATGGCCATGGAGGCCATGGAGGACGCGCTGCTCGG | 1465 |
| MYC-*Mtl5*-Rv | ATCTAGAGTCGCGGCCGCACTACTCAATTTTCAGCCCCT |  |
| GFP-*Lin9*-Fw | TCGAGCGGTGGAGCTGCAGGAATGCACCGGGGCGGGCAGCC | 1677 |
| GFP*-Lin9*-Rv | GATCTAGAGTCGCGGCCGCTTCAGTCTCTGTTCGTGTTGT |  |
| GFP-*Lin37*-Fw | TCGAGCGGTGGAGCTGCAGGAATGTTCCCGGTAAAGGTGAA | 741 |
| GFP-*Lin37*-Rv | GATCTAGAGTCGCGGCCGCTTCACTGCCGGTCATACATCT |  |
| GFP-*Lin52*-Fw | TCGAGCGGTGGAGCTGCAGGAATGGGCTGGAAGATGGCGTC | 351 |
| GFP-*Lin52*-Rv | GATCTAGAGTCGCGGCCGCTCTACTTCTTGGGCTTTTCTA |  |
| GFP-*Rbbp4*-Fw | TCGAGCGGTGGAGCTGCAGGAATGGCTGACAAGGAAGCGGC | 1278 |
| GFP-*Rbbp4*-Rv | GATCTAGAGTCGCGGCCGCTCTAGGACCCTTGTCCCTCTG |  |
| GFP-*Mybl1*-Fw | TCGAGCGGTGGAGCTGCAGGAATGGCGAAGAGGTCGCGCAG | 2256 |
| GFP-*Mybl1*-Rv | GATCTAGAGTCGCGGCCGCTTTAGAGTATTAGAGCTCTTG |  |
| GFP-*Lin54*-Fw | TCGAGCGGTGGAGCTGCAGGAATGGAGGTGGTGCCGGCCGA | 2250 |
| GFP-*Lin54*-Rv | GATCTAGAGTCGCGGCCGCTTTAGCAGTGCATGGCACAGG |  |
| p-N1-BB-linker-Fw | AGCGGCCGCGACTCTAGATC |  |
| p-N1-BB-linker-Rv | TCCTGCAGCTCCACCGCTCGACTTGTACAGCTCGTCCATGC |  |
| MYC-*Mtl5*-ΔC1-Fw | GCATTGCTTGCAAAAACTATTCCTGGGAAGTAGTGGAGGC | 1321 |
| MYC-*Mtl5*-ΔC1-Rv | ATAGTTTTTGCAAGCAATGC |  |
| MYC-*Mtl5*-ΔC2-Fw | GGCAGGCCTTCTCCTGTATCTCCCCAAGCTTGGCTGAGCA | 1393 |
| MYC-*Mtl5*-ΔC2-Rv | GATACAGGAGAAGGCCTGCC |  |
| MYC-*Mtl5*-ΔC3-Fw | TGCGGCCGCGACTCTAGAT | 1366 |
| MYC-*Mtl5*-ΔC3-Rv | ATCTAGAGTCGCGGCCGCACTAACAGTGCTCCTGCTCTG |  |
| CMV-Myc-S-Fw | TTCTTCCTTTTCCCCACCCC |  |
| CMV-Myc-L-Rv | GGGGTGGGGAAAAGGAAGAA |  |
| BD-MTL5-Fw | ATGGCCATGGAGGCCGAATTCATGGAGGACGCGCTGCTCGG | 1428 |
| BD-MTL5-Rv | CCGCTGCAGGTCGACGGATCCCTACTCAATTTTCAGCCCCT |  |
| AD-MTL5-Fw | GCCATGGAGGCCAGTGAATTCATGGAGGACGCGCTGCTCGG | 1428 |
| AD-MTL5*-*Rv | CAGCTCGAGCTCGATGGATCCCTACTCAATTTTCAGCCCCT |  |
| BD-LIN9-Fw | ATGGCCATGGAGGCCGAATTCATGCACCGGGGCGGGCAGCC | 1677 |
| BD-LIN9-Rv | CCGCTGCAGGTCGACGGATCCTCAGTCTCTGTTCGTGTTGT |  |
| BD-LIN37-Fw | ATGGCCATGGAGGCCGAATTCATGTTCCCGGTAAAGGTGAA | 741 |
| BD-LIN37-Rv | CCGCTGCAGGTCGACGGATCCTCACTGCCGGTCATACATCT |  |
| BD-LIN52-Fw | ATGGCCATGGAGGCCGAATTCATGGGCTGGAAGATGGCGTC | 351 |
| BD-LIN52-Rv | CCGCTGCAGGTCGACGGATCCCTACTTCTTGGGCTTTTCTA |  |
| BD-LIN54-Fw | ATGGCCATGGAGGCCGAATTCATGGAGGTGGTGCCGGCCGA | 2250 |
| BD-LIN54-Rv | CCGCTGCAGGTCGACGGATCCTTAGCAGTGCATGGCACAGG |  |
| BD-RBBP4-Fw | ATGGCCATGGAGGCCGAATTCATGGCTGACAAGGAAGCGGC | 1278 |
| BD-RBBP4-Rv | CCGCTGCAGGTCGACGGATCCCTAGGACCCTTGTCCCTCTG |  |
| BD-MYBL1-Fw | ATGGCCATGGAGGCCGAATTCATGGCGAAGAGGTCGCGCAG | 2256 |
| BD-MYBL1-Rv | CCGCTGCAGGTCGACGGATCCTTAGAGTATTAGAGCTCTTG |  |
| BD-LIN9-N-Fw | ATGGCCATGGAGGCCGAATTCATGCACCGGGGCGGGCAGCCTTTGAAAAAG | 453 |
| BD-LIN9-N-Rv | CCGCTGCAGGTCGACGGATCCCTATGGCTTGTCTATGTTTG |  |
| BD-LIN9-M-Fw | ATGGCCATGGAGGCCGAATTCATGCTTTTTGAAGGAGATAA | 576 |
| BD-LIN9-M-Rv | CCGCTGCAGGTCGACGGATCCCTATCTCCAAGGTGACTGCC |  |
| BD-LIN9-C-Fw | ATGGCCATGGAGGCCGAATTCATGAGTAAAGTTTCTGGCTC | 660 |
| BD-LIN9-C-Rv | CCGCTGCAGGTCGACGGATCCTCAGTCTCTGTTCGTGTTGT |  |
| AD-MTL5-N-Fw | GCCATGGAGGCCAGTGAATTCATGGAGGACGCGCTGCT | 753 |
| AD-MTL5-N-Rv | CAGCTCGAGCTCGATGGATCCCTATCCATTATCAACCTGTG |  |
| AD-MTL5-M-Fw | GCCATGGAGGCCAGTGAATTCATGGCTCTCCCATCAGCTGT | 366 |
| AD-MTL5-M-Rv | CAGCTCGAGCTCGATGGATCCCTAATAGTTTTTGCAAGCAA |  |
| AD-MTL5-C-Fw | GCCATGGAGGCCAGTGAATTCATGGAAGAAAGTCCAGAACG | 321 |
| AD-MTL5-C-Rv | CAGCTCGAGCTCGATGGATCCCTACTCAATTTTCAGCCCCT |  |
| AD-MTL5-C1-Rv | CAGCTCGAGCTCGATGGATCCCTAGATACAGGAGAAGGCCTGCC | 147 |
| AD-MTL5-C2-Fw | GCCATGGAGGCCAGTGAATTCATGTCCTGGGAAGTAGTGGAGGC | 72 |
| AD-MTL5-C2-Rv | CAGCTCGAGCTCGATGGATCCCTAACAGTGCTCCTGCTCTGCTT |  |
| AD-MTL5-C3-Fw | GCCATGGAGGCCAGTGAATTCATGTCCCCAAGCTTGGCTGAGCA | 102 |
