# Supplementary material for: Nuclear translocation of MTL5 from cytoplasm requires its direct interaction with LIN9 and is essential for male meiosis and fertility
Source: PLoS Genet. 2021 Aug 13;17(8):e1009753. doi: 10.1371/journal.pgen.1009753 (PMC8386835; doi:10.1371/journal.pgen.1009753)
Supplement: S3 Table — (DOCX) [file pgen.1009753.s016.docx]

| **Table S3. Antibodies used in this study**   \| **Antibodies** \| **Host** \| **Company** \| **Catalog number** \| **Dilution** \| \| \| --- \| --- \| --- \| --- \| --- \| --- \| \| SYCP3 \| Rabbit \| Novus \| NB300-232 \| \| 1:5000 \| \| SYCP3 \| Mouse \| Abcam \| ab97672 \| \| 1:200 \| \| H1t \| Guinea pig \| Gift from Mary Ann Handel,  Jackson lab \| \| \| 1:500 \| \| GFP \| Mouse \| Abmart \| M20004 \| \| 1:100 (IF)  1:2000 (WB) \| \| β-Actin \| Rabbit \| Abcam \| ab8227 \| \| 1:3000 \| \| MYC tag \| Mouse \| Santa Cruz \| sc-40 \| \| 1:500 \| \| Rabbit IgG \| Rabbit \| ABclonal \| AC005 \| \| 2 ug per IP \| \| MTL5-N \| Rabbit \| YNK Biotech, Suzhou \| Home-made \| \| 1:300-500 (IF)  1:1000 (WB) \| \| MTL5-N \| Rat \| ABclonal, Wuhan \| Home-made \| \| 1:500 (WB) \| \| MTL5-C \| Rabbit \| YNK Biotech, Suzhou \| Home-made \| \| 1:500-1:1000 (WB) \| \| LIN9 \| Rabbit \| Novus \| NBP1-80690 \| \| 1:100 (IF) \| \| LIN9 \| Rabbit \| Proteintech \| 17882-1-AP \| \| 1:500 (WB) \| \| RPA2 \| Rabbit \| ABclonal \| A2189 \| \| 1:100 \| \| DMC1 \| Guinea pig \| ABclonal \| Home-made \| \| 1:100 \| \| Lectin PNA \|  \| Thermo Fisher \| L21409, AF488 conjugate \| \| 1:200 \| \| Donkey anti-rabbit IgG (H+L) (AF555) \| Secondary antibody \| Molecular Probes \| A31572 \| \| 1:200 \| \| Goat anti-mouse IgG1 (AF488) \| Secondary antibody \| Molecular Probes \| A21121 \| \| 1:100 \| \| Fluorescein(FITC)-conjugated Affinipure Donkey Anti-Guinea Pig IgG(H+L) \| Secondary antibody \| Jackson ImmunoResearch \| 706-095-148 \| \| 1:100 \| \| Goat Anti-Guinea Pig IgG(H+L) 555 \| Secondary antibody \| Thermo Fisher \| A-21435 \| \|  \| \| HRP Donkey anti-Rabbit IgG \| Secondary antibody \| BioLegend \| 406401 \| \| 1:10000 \| \| HRP Goat anti-Mouse IgG \| Secondary antibody \| BioLegend \| 405306 \| \| 1:10000 \| \| HRP Goat anti-rat IgG \| Secondary antibody \| BioLegend \| 405405 \| \| 1:10000 \| |
| --- | --- | --- | --- | --- | --- | --- | --- | --- | --- | --- | --- | --- | --- | --- | --- | --- | --- | --- | --- | --- | --- | --- | --- | --- | --- | --- | --- | --- | --- | --- | --- | --- | --- | --- | --- | --- | --- | --- | --- | --- | --- | --- | --- | --- | --- | --- | --- | --- | --- | --- | --- | --- | --- | --- | --- | --- | --- | --- | --- | --- | --- | --- | --- | --- | --- | --- | --- | --- | --- | --- | --- | --- | --- | --- | --- | --- | --- | --- | --- | --- | --- | --- | --- | --- | --- | --- | --- | --- | --- | --- | --- | --- | --- | --- | --- | --- | --- | --- | --- | --- | --- | --- | --- | --- | --- | --- | --- | --- | --- | --- | --- | --- | --- | --- | --- | --- | --- | --- | --- | --- | --- | --- | --- | --- | --- | --- | --- | --- | --- | --- | --- | --- | --- | --- | --- | --- | --- | --- |
